# Time-resolved single-cell transcriptomics reveals the landscape and dynamics of hepatic cells in sepsis-induced acute liver dysfunction

**DOI:** 10.1016/j.jhepr.2023.100718

**Published:** 2023-03-01

**Authors:** Gan Chen, Chao Ren, Yao Xiao, Yujing Wang, Renqi Yao, Quan Wang, Guoxing You, Mingzi Lu, Shaoduo Yan, Xiaoyong Zhang, Jun Zhang, Yongming Yao, Hong Zhou

**Affiliations:** 1 Institute of Health Service and Transfusion Medicine, Academy of Military Medical Sciences, Beijing, China; 2 Translational Medicine Research Center, Fourth Medical Center and Medical Innovation Research Division of the Chinese PLA General Hospital, Beijing, China; 3 Department of Pulmonary and Critical Care Medicine, Beijing Chaoyang Hospital, Capital Medical University, Beijing, China; 4 Beijing Science and Technology Innovation Research Center, Beijing, China

**Keywords:** Sepsis, Single-cell RNA sequencing, Acute liver dysfunction, Activating transcription factor 4

## Abstract

**Background & Aims:**

Sepsis-induced acute liver dysfunction often occurs early in sepsis and can exacerbate the pathology by triggering multiple organ dysfunction and increasing lethality. Nevertheless, our understanding of the cellular heterogeneity and dynamic regulation of major nonparenchymal cell lineages remains unclear.

**Methods:**

Here, single-cell RNA sequencing was used to profile multiple nonparenchymal cell subsets and dissect their crosstalk during sepsis-induced acute liver dysfunction in a clinically relevant polymicrobial sepsis model. The transcriptomes of major liver nonparenchymal cells from control and sepsis mice were analysed. The alterations in the endothelial cell and neutrophil subsets that were closely associated with acute liver dysfunction were validated using multiplex immunofluorescence staining. In addition, the therapeutic efficacy of inhibiting activating transcription factor 4 (ATF4) in sepsis and sepsis-induced acute liver dysfunction was explored.

**Results:**

Our results present the dynamic transcriptomic landscape of major nonparenchymal cells at single-cell resolution. We observed significant alterations and heterogeneity in major hepatic nonparenchymal cell subsets during sepsis. Importantly, we identified endothelial cell (CD31^+^Sele^+^Glut1^+^) and neutrophil (Ly6G^+^Lta4h^+^Sort1^+^) subsets that were closely associated with acute liver dysfunction during sepsis progression. Furthermore, we found that ATF4 inhibition alleviated sepsis-induced acute liver dysfunction, prolonging the survival of septic mice.

**Conclusions:**

These results elucidate the potential mechanisms and subsequent therapeutic targets for the prevention and treatment of sepsis-induced acute liver dysfunction and other liver-related diseases.

**Impact and Implications:**

Sepsis-induced acute liver dysfunction often occurs early in sepsis and can lead to the death of the patient. Nevertheless, the pathogenesis of sepsis-induced acute liver dysfunction is not yet clear. We identified the major cell types associated with acute liver dysfunction and explored their interactions during sepsis. In addition, we also found that ATF-4 inhibition could be invoked as a potential therapeutic for sepsis-induced acute liver dysfunction.

## Highlights

• scRNA-seq reveals the cellular composition and dynamic transcriptomic landscape of major liver nonparenchymal cells in sepsis-induced acute liver dysfunction.
• scRNA-seq analysis and immunofluorescence identify the endothelial cell (CD31^+^Sele^+^Glut1^+^) and neutrophil (Ly6G^+^Lta4h^+^Sort1^+^) subsets that were closely associated with acute liver dysfunction.
• The ATF4 pathway activation is a potential mechanism contributing to sepsis-induced acute liver dysfunction, and ATF4 inhibition alleviates sepsis-induced acute liver dysfunction and prolongs the survival of septic mice.

## Graphical abstract

**Figure:**
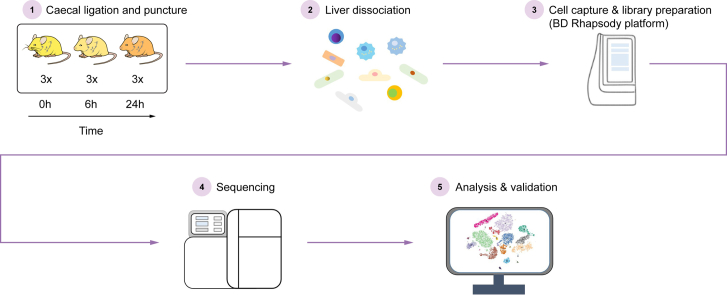

## Introduction

Sepsis is characterised by a dysregulated host immune response to trauma or overwhelming infection (*e.g.* severe COVID-19 infection^1^), leading to life-threatening organ dysfunction.^2^ Despite numerous advances in medical care, sepsis remains the most common cause of death in intensive care units at present.^3^ Acute liver dysfunction often occurs in the early stage of sepsis and has a significant effect on the severity and prognosis of sepsis. To date, specific therapeutic measures in current clinical practice, to resolve sepsis-induced acute liver dysfunction other than liver transplantation, is still lacking.^4^ However, the shortage of liver sources as well as lifelong immunosuppression and a high medical cost associated with liver transplantation has resulted in an urgent need for alternative therapeutic interventions that is effective in treating sepsis-induced acute liver dysfunction.

Clarifying the pathogenesis of sepsis-induced acute liver dysfunction will provide opportunities to develop novel therapeutic interventions. Although previous studies have reported that acute liver dysfunction involves a complex interplay between multiple nonparenchymal cell lineages including neutrophils, endothelial cells, and Kupffer cells,^5^ their cellular heterogeneity and dynamic regulation leading to acute liver dysfunction remain poorly understood.

The rapid development of single-cell RNA sequencing (scRNA-seq) provides high-dimensional information about tissues and an unprecedented understanding of cellular composition, response, and crosstalk in a pathological state.^6^^,^^7^ The scRNA-seq has been performed to study several liver diseases from mice and humans.^6^^,^^8^^,^^9^ However, most of these studies have focused on chronic liver diseases including liver cirrhosis, non-alcoholic fatty liver disease, and liver cancer,^8^^,^^10^ and not many have been conducted on acute liver diseases.

Therefore, in this study, we performed a scRNA-seq experiment on livers following sepsis-induced acute liver dysfunction in a clinically relevant polymicrobial sepsis model. Our analyses revealed the major cell types associated with acute liver dysfunction including neutrophils, endothelial cells, and Kupffer cells; identified subclusters of each cell type; and uncovered their dynamic transformations and interactions. Notably, we reported the extensive activating transcription factor 4 (ATF4) activation in major hepatic cells, as well as the therapeutic efficacy of ATF4 inhibition for sepsis and sepsis-induced acute liver dysfunction.

## Materials and methods

### Animal care, sepsis model, and animal experiment

All experiments and procedures conformed to the National Institutes of Health guidelines, with the approval of the Institutional Animal Care and Use Committee of the Academy of Military Medical Sciences. The male C57BL6/J mice (7–8 weeks) were obtained from Beijing Vital River Laboratories (Beijing, China) and housed according to the following standard laboratory procedures. After a minimum of 3 days of acclimatisation, the caecal ligation and puncture (CLP) sepsis model was induced as described previously.^11^ Briefly, mice were anesthetised with pentobarbital sodium (50 mg/kg). After sterilisation, a midline laparotomy was conducted to expose the caecum, which was ligatured and perforated with a sterile 21-gauge needle. Then a small droplet of faeces was extruded from the puncture, and the caecum was returned into the peritoneal cavity. Subsequently, the mice were resuscitated via a s.c. administration of 1 ml of normal saline.

Nine mice were randomly allocated to the control or sepsis groups; the normal group (control check [CK], n = 3) acted as the control group, whereas the sepsis groups were subjected to CLP and sacrificed at 6 (n = 3) or 24 h (n = 3). The livers and blood from mice in the three groups were immediately collected for further processing.

### Cell isolation

After being washed twice with cold normal saline, liver tissues from the same group were pooled and minced to <0.5-mm cubic pieces, followed by enzymatic digestion (1 h at 37 °C) with manual shaking every 5 min. After filtering, dead cell removal, and lysis of red blood cells, the hepatic cells were counted using BD Rhapsody™ Scanner (BD Biosciences, San Jose, CA, USA).

### Single-cell RNA sequencing

Single-cell libraries were prepared using the BD Rhapsody Single-Cell Analysis System (BD Biosciences) following the manufacturer’s guidelines. Libraries were sequenced using multiple runs on an Illumina NovaSeq 6000 platform (Illumina, San Diego, CA, USA) in a 2 × 150 bp paired-end mode.

### Sequencing data analysis

The obtained RNA-sequencing data were processed into the expression matrix Fastq via the BD Rhapsody Analysis Pipeline (v1.9). After quality control, normalisation, and batch correction using the R package ‘Seurat’ and fastMNN, BD DataView software (BD Biosciences) and the R package ‘Seurat’ (v3.1.1) were used to analyse the expression matrix.

To reduce the dimensionality of all data, principal component analysis was conducted in Seurat. For t-distributed stochastic neighbour embedding (tSNE) projection and clustering analysis, the cells were clustered based on a graph-based clustering approach and visualised in two dimensions using tSNE. The FindAllMarkers function in Seurat was used to identify the marker genes of each cluster, and the FindMarkers function was used to analyse the differentially expressed genes. A *p* value <0.05 and |log_2_ fold change| >0.58 were set as thresholds for significantly differential expression.

The Kyoto Encyclopedia of Genes and Genomes (KEGG) analysis was performed using the R package ‘enrichplot’. Cell–cell interaction analysis was performed using the CellChat R package (v1.1.3).

Single-cell regulatory network inference and clustering (SCENIC) analysis was conducted using the motif database for RcisTarget and GRNboost (SCENIC v1.1.2.2, which corresponds to AUCell v1.4.1 and RcisTarget v1.2.1). The ‘RcisTarget’ package was used to identify transcription factor (TF) binding motifs and potential target genes (regulons). The activity of the regulon group in each cell was classified using the ‘AUCell’ package. The regulon specificity scores for each cell type were calculated using the ‘scFunctions’ package.

### Drug administration

To evaluate the therapeutic effect of integrated stress response inhibitor (ISRIB), a total of 28 mice were randomly divided into the control or experimental groups; the normal group (CK, n = 3) and the ISRIB-treated group (5 mg/kg, n = 3) acted as controls, whereas the experimental groups were i.p. treated with ISRIB (5 mg/kg, n = 11) or an equal volume of vehicle (n = 11) 2 h before CLP. Mice were sacrificed 24 h after CLP, and the livers and plasma were collected and stored until assayed.

### Blood biochemistry and bloodocyte analysis

Blood biochemistry was analysed using a biochemical auto analyser (Pointcare V2, MNCHIP, Tianjin, China). Complete blood counts were analysed using a BC-5000 Vet auto haematology analyser (Mindray, Shenzhen, China).

### Lipid peroxidation, neutrophil infiltration, and IL-6 content in livers

The livers were homogenised in ice-cold saline (Shijiazhuang Siyao Ltd., Hebei, China) and centrifuged (1000×*g*, 6 min, 4 °C). The obtained homogenates were then assayed for malondialdehyde (MDA) content, myeloperoxidase (MPO) activity, and IL-6 level, according to the manufacturer’s instructions, as described previously.^12^

### Histological analysis and immunofluorescence

The paraformaldehyde-fixed livers were dehydrated, embedded in paraffin, and cut into 5- to 7-μm-thick sections before being subjected to H&E staining as described previously.^12^ The severity level of liver damage was assessed for inflammatory infiltration, cell swelling, and tissue architecture disruption in a blinded fashion and scored on a 4-point scale (0, none; 1, slight; 2, moderate; 3, severe).

For immunofluorescence assays, the liver sections were incubated with anti-CD31 (1:3,000, Proteintech, IL, USA), anti-Glut1 (1:100, Proteintech), anti-Sele (1:2,000, Proteintech), anti-Ly6G (1:3,000, Servicebio, Wuhan, China), anti-Lta4h (1:1,000, Proteintech), anti-Sort1 (1:400, Proteintech), anti-ATF4 (1:1,000, ABclonal, Wuhan, China), anti-Fosl1 (1:1,000, ABclonal), anti–NF–κB1(1:1,000, ABclonal), and anti-F4/80 (1:500, Servicebio) primary antibodies, followed by washing and incubation with the fluorophore-labelled secondary antibody, and visualisation using a confocal microscope (Nikon, Tokyo, Japan).

### Survival experiments

A total of 100 mice were randomised into the normal control or sepsis model groups. The sepsis model groups were i.p. treated with ISRIB (8 mg/kg, n = 45) or an equivalent volume of vehicle (n = 45) 2 h before CLP. The normal mice acted as the control group.

### Statistical analysis

Data are shown as mean ± SEM. Statistical differences between groups were determined using one-way ANOVA or unpaired Student’s *t* test. Survival data were analysed using the log-rank test. ∗*p* <0.05, ∗∗*p* <0.01, and ∗∗∗*p* <0.001 were considered the thresholds for statistical significance of differences.

## Results

### scRNA-seq identified multiple cell populations in the liver from control and sepsis mice

**Fig. 1:**
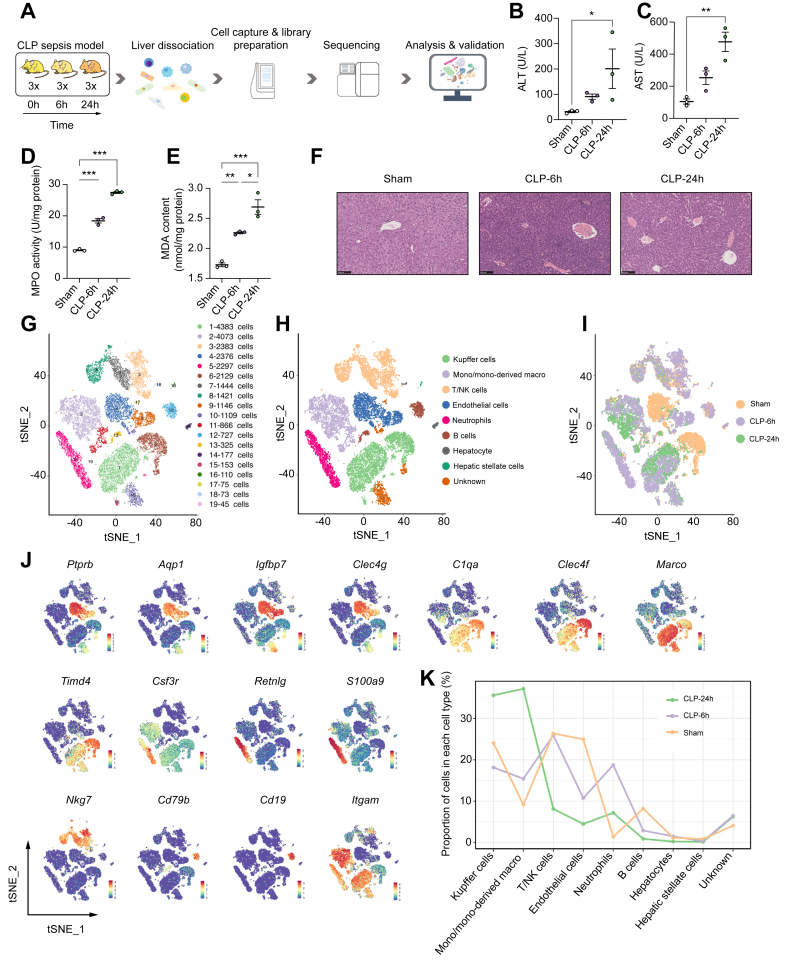
scRNA-seq identified hepatic cell populations and specific markers in the mice liver. (A) Schematic diagram indicating the procedure for scRNA-seq. For each experimental condition, the hepatic cells were pooled from three mice in each group. (B) The levels of ALT in plasma. (C) The levels of AST in plasma. (D) MPO activity and (E) the MDA content in the liver. (F) Histological injury of liver (scale bar, 100 μm). (G) UMAP plot of hepatic cells, with colours denoting different cell type clusters. (H) UMAP plot of hepatic cells, with colours denoting different clusters. (I) UMAP plot of cell clusters in hepatic cells across the indicated conditions. (J) The markers indicating group identities. (K) The proportion of hepatic cell populations in each sample. ∗*p* <0.05; ∗∗*p* <0.01; ∗∗∗*p* <0.001. Statistical differences between groups were assessed using one-way ANOVA for (B)–(E). ALT, alanine aminotransferase; AST, aspartate aminotransferase; CLP, caecal ligation and puncture; CLP-24 h, 24 h after CLP; CLP-6h, 6 h after CLP; MDA, malondialdehyde; MPO, myeloperoxidase; scRNA-seq, single-cell RNA sequencing; T/NK, T/natural killer; tSNE, t-distributed stochastic neighbour embedding; UMAP, uniform manifold approximation and projection.

To elucidate diverse hepatic nonparenchymal cell types and comprehensively characterise their dynamic changes during the pathological progression of sepsis-induced acute liver dysfunction, we performed scRNA-seq analysis of the livers at different time points (6 and 24 h) after sepsis using the BD Rhapsody platform (Fig. 1A). These two time points reflected the major stages in the development of sepsis-induced acute liver dysfunction, corresponding to a progressive increase in liver injury. As shown in Fig. 1B and C, the plasma levels of alanine aminotransferase (ALT) and aspartate aminotransferase (AST), which are used as biomarkers for acute liver dysfunction,^13^ gradually became elevated over time. Similarly, MDA content, MPO activity, and histological injury in the liver also increased gradually with time, indicating that sepsis could induce lipid peroxidation and neutrophil infiltration during its pathological progression (Fig. 1D–F). In addition, the sepsis model mice also exhibited acute kidney injury and had a low level of glucose and total protein in blood (Fig. S1A–D).

A total of 27,972 liver nonparenchymal cells and hepatocytes from control and sepsis mice were further analysed after passing quality control metrics and having corrected for batch effect. Eight major cellular clusters consisting of 19 clusters (marker genes shown in Fig. S1F) were identified (Fig. 1G–J), including endothelial cells (*Ptprb*, *Aqp1*, *Igfbp7*, *Clec4g*, *Ehd3*, *Ushbp1*, *Oit3*, *Il1a*, *F8*, *Bmp2*, *C1qtnf1*, *Mmrn2*, *Pcdh12*, and *Dpp4*), Kupffer cells (*C1qa*, *C1qb*, *C1qc*, *Clec4f*, *Csf1r*, *Adgre1*, *Clec4f*, *Irf7*, *Spic*, *Timd4*, and *Marco*), neutrophils (*Csf3r*, *Retnlg*, *S100a8*, *S100a9*, *Slpi*, *Mmp9*, *Mmp8*, and *Adam8*), T/natural killer cells (*Nkg7*), B cells (*Igkc*, *Cd22*, *Cd79b*, *Cd19*, *Cd79a*, *Ebf1*, and *Pax5*), monocytes/monocyte-derived macrophages (*Itgam* and *Ccr2*), hepatocytes (*Alb*, *Cyp2e1*, *Apob*, *Asgr1*, *Pck1*, *Hp*, *Ass1*, and *Apoe*), and hepatic stellate cells (*DCN*, *Hgf*, *Col14a1*, *Col1a1*, *Col1a2*, *Col3a1*, *Colec11*, *Cxcl12*, and *Cygb*).

In addition, the relative composition of different cell types across different stages of disease and healthy controls was investigated to uncover substantial changes during disease progression. A rapid increase of the neutrophil population was observed 6 h after CLP when compared with that of controls. Subsequently, the proportion of neutrophils declined at 24 h after CLP compared with that at 6 h after CLP (Fig. 1K). The relative proportion of the endothelial and B cells decreased with pathological progression, whereas that of monocytes/monocyte-derived macrophages increased with pathological progression. In addition, there was a slight decrease in the proportion of Kupffer cell population at 6 h after CLP compared with that of controls, but this proportion robustly increased at 24 h after CLP, which indicated a rapid supplement of Kupffer cells from blood circulation. These results suggested that different hepatic nonparenchymal cell types, especially immune populations changed dynamically at different time points and reacted collectively to acute liver dysfunction via distinct functions.

Pecam1(CD31), Adgre1(F4/80), and Ly6g have been reported as cell makers of endothelial cells, Kupffer cells, and neutrophils, respectively.[14], [15], [16], [17] The tSNE plots for *Pecam1*, *Ly6g*, and *Adgre1* expressions in all cell clusters are shown in Fig. S1E. Our results show that regions with high *Ly6g*, *Pecam1*, and *Adgre1* expression are strongly correlated with the neutrophils, endothelial cells, and Kupffer cells, respectively.

### scRNA-seq revealed disease-specific endothelial cell subpopulations

Previous studies have highlighted liver endothelial cells that orchestrate the progression of liver injury.^8^^,^^18^ Thus, further analysis on our scRNA-seq data was performed to investigate the transcriptional diversity of endothelial cells in liver.

**Fig. 2:**
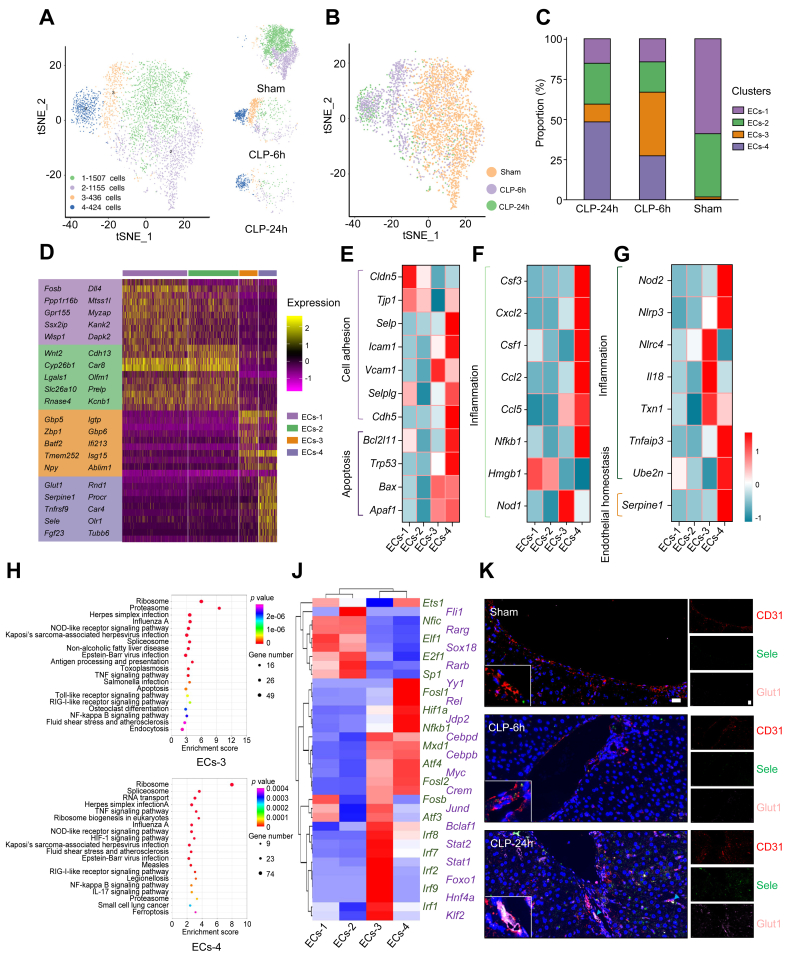
Hepatic endothelial cell dynamics and functionality in healthy and septic livers. (A) Subclustering of endothelial cells in healthy and septic livers. (B) UMAP plot of endothelial cells across the indicated conditions. (C) The proportion of endothelial cell subclusters in each sample. (D) Heatmap plots demonstrating expression of specified genes among endothelial cell subclusters. (E–G) Heatmap showing gene expression by the indicated endothelial cell subclusters. (H) The enriched pathway associated with ECs-3 and ECs-4. Colours indicate significance of enrichment, and circle sizes indicate number of genes falling into respective categories. (J) Subpopulation-specific regulons of each subpopulation revealed by SCENIC analysis. (K) Immunofluorescence staining results showing the spatial distribution of the ECs-4 subpopulations in healthy and septic mouse livers. Scale bars, 20 μm. CLP, caecal ligation and puncture; CLP-24 h, 24 h after CLP; CLP-6h, 6 h after CLP; ECs-1 to ECs-4, endothelial cell subclusters 1 to 4; NOD, nucleotide oligomerisation domain; SCENIC, single-cell regulatory network inference and clustering; TNF, tumour necrosis factor; tSNE, t-distributed stochastic neighbour embedding; UMAP, uniform manifold approximation and projection.

The liver endothelial cells were grouped into four subclusters (Fig. 2A and B); ECs-1 and ECs-2 accounted for up to 98.44% of liver endothelial cells under healthy conditions (Fig. 2C), whereas endothelial cells in ECs-3 and ECs-4 were nearly exclusively present in the livers of sepsis mice, and the proportion of endothelial cells in ECs-4 increased with the pathological progression of sepsis-induced acute liver dysfunction (Fig. 2C), indicating that the ECs-4 subcluster represented a unique population of endothelial cells associated with the pathogenesis of sepsis-induced acute liver dysfunction. Based on a subsequent gene enrichment analysis, ECs-3 was found differentially expressing *Zbp1*, *Batf2*, and *Isg15*. Zbp1 can act as a DNA sensor and mediate the recruitment of RIP1 and RIP3 to activate the NF-κB and NLRP3 inflammasome pathways.^19^^,^^20^

In contrast, ECs-4 showed the highest expression of *Glut1*, *Serpine1*, *Sele*, *Fgf23*, *Car4*, *Rnd1*, and *Tnfrsf9* (Fig. 2D and Fig. S2C). Among these highly expressed genes of ECs-4, the expression of *Serpine1*, *Sele*, and *Fgf23* is associated with endothelial dysfunction.^18^^,^^21^^,^^22^ *Tnfrsf9* expression could exacerbate inflammation through promoting cell adhesion and leucocyte chemoattraction.^22^ Similarly, the increase of *Rnd1* expression could induce actin cytoskeletal rearrangement of endothelium, thereby aggravating the inflammatory response.^23^ This evidence further indicates that the dysfunction of ECs-4 leads to the acceleration of sepsis-induced acute liver dysfunction.

In addition, ECs-3 showed an upregulation of inflammation-associated genes (*Il18*, *Nlrc4*, *Txn1*, and *Nod1*) (Fig. 2F and G). As for cell adhesion molecules, ECs-3 exhibited a relatively high expression of *Vcam-1* and a low expression of *Tjp1* and *Cldn5* (Fig. 2E), indicating poor cellular adhesion of endothelial cells in ECs-3. The ECs-4 subcluster also showed an upregulation of genes related to inflammation (*Nlrp3*, *Tnfaip3*, *Ube2n*, *Cxcl2*, *Ccl2*, *Ccl5*, *Csf3*, *Nfkb1*, and *Nod2*) (Fig. 2F and G), cell adhesion (*Cdh5*, *Selplg*, *Icam1*, and *Selp*) (Fig. 2E), and apoptos*is* (*Apaf1*, *Bax*, *Trp53*, and *Bcl2l11*) (Fig 2E)*.* Interestingly, ECs-4 exhibited a low level of *Hmgb1* (Fig. 2F)*.* Hmgb1 is considered an important mediator in the pathogenesis of sepsis-induced acute liver dysfunction.^24^^,^^25^ KEGG pathway analyses revealed that the genes that were upregulated in ECs-3 belonged to the Toll-like receptor, antigen processing and presentation, and nucleotide oligomerisation domain (NOD)-like receptor signalling pathways (Fig. 2H), whereas the genes that were upregulated in ECs-4 belonged to the tumour necrosis factor (TNF)/NF-κB, IL-17, and NOD-like receptor signalling pathways (Fig. 2H and Fig. S2A and B).

Furthermore, SCENIC analysis was used to assess the expression status of TFs in different endothelial cell subpopulations. As shown in Fig. 2J, the genes regulated by the Yy1, Fosl1, Hif1α, and Atf4 TFs were upregulated in ECs-4. Notably, Atf4 is involved in the complex process of cellular stress response^26^^,^^27^ and can be used as a pharmacological target in several diseases including diabetes mellitus,^28^ atrial fibrillation,^29^ and age-related memory decline.^30^ High endothelial Hif-1α expression promoted CXCL1 expression and monocyte adhesion to endothelial cells,^31^ and Yy1 expression was correlated with the inflammatory NK-κB activity and neutrophil infiltration and subsequently resulted in enhanced inflammatory effects.^32^^,^^33^ Fosl1 is part of the activator protein complex, which makes up the TF activator protein 1 (AP-1). AP-1 has been suggested to be responsible for activating inflammation.^34^

To confirm the emergence of ECs-4 during the pathogenesis of sepsis-induced acute liver dysfunction, the immunofluorescence staining was performed using CD31, Sele, and Glut1 antibodies to detect this endothelial cell subpopulation (Fig. 2K). Consistent with single-cell analysis, the proportion of CD31^+^Sele^+^Glut1^+^ endothelial cells increased gradually, concomitant with the development of acute liver dysfunction. In addition, total endothelial cells were detected using CD31 immunofluorescence staining. Our results indicate that the number of total endothelial cells decrease with pathological progression, being consistent with the scRNA-seq result (Fig. S2D).

Altogether, the analysis of endothelial cells indicated that hepatic endothelial cells exhibited heterogeneity, and differences among subtypes with distinct gene expression patterns represented their unique functionality during sepsis. Here, the unique subpopulation of endothelial cells with endothelial dysfunction-related genetic characteristics was also identified and is speculated to be closely related to the pathology of sepsis-induced acute liver dysfunction.

### scRNA-seq revealed heterogeneity of neutrophils in liver during sepsis

Neutrophils, which are well characterised to be recruited rapidly at sites of infection, function as first-line responders specialised in elimination of invading pathogens. However, simultaneously exaggerated activation and uncontrolled tissue infiltration of neutrophils cause excess oxidative stress, inflammation, and subsequent tissue injury,^35^ thus making them a potential therapeutic target to treat sepsis in our study and others.^13^^,^^36^ However, neutrophil populations in sepsis are not homogenous; therefore, exploring neutrophil heterogeneity will help regulate neutrophils accurately to obtain a balance between protective immunity and tissue injury.

**Fig. 3:**
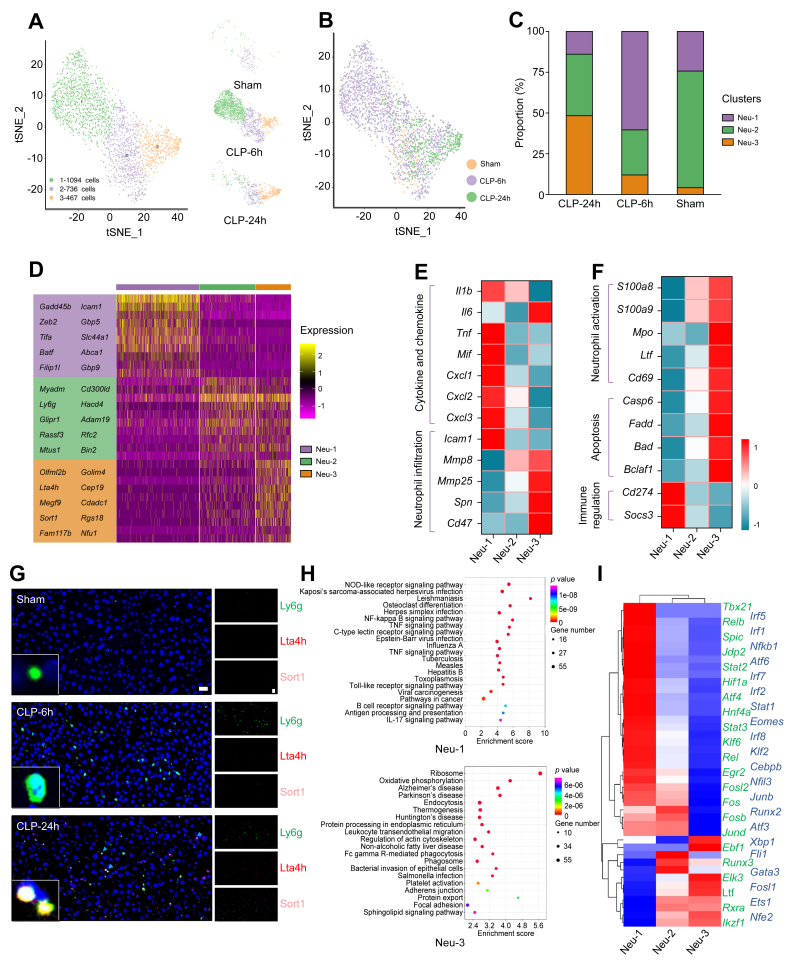
Hepatic neutrophil dynamics and functionality in healthy and septic livers. (A) Subclustering of neutrophils in healthy and septic livers. (B) UMAP plot of neutrophils across the indicated conditions. (C) The proportion of neutrophil subclusters in each sample. (D) Heatmap plots demonstrating expression of specified genes among neutrophil subclusters. (E and F) Heatmap showing gene expression by the indicated neutrophil subclusters. (G) Immunofluorescence staining results showing the spatial distribution of the Neu-3 subpopulations in healthy and septic mouse livers. Scale bars, 20 μm. (H) The enriched pathway associated with Neu-1 and Neu-3. Colours indicate significance of enrichment and circle sizes indicate number of genes falling into respective categories. (I) Subpopulation-specific regulons of each subpopulation revealed by SCENIC analysis. CLP, caecal ligation and puncture; CLP-24 h, 24 h after CLP; CLP-6h, 6 h after CLP; Neu-1 to Neu-3, neutrophil subclusters 1 to 3; NOD, nucleotide oligomerisation domain; SCENIC, single-cell regulatory network inference and clustering; TNF, tumour necrosis factor; tSNE, t-distributed stochastic neighbour embedding; UMAP, uniform manifold approximation and projection.

The hepatic neutrophils were further grouped into three subclusters: Neu-1 (mainly expressing *Gadd45b*, *Icam1*, *Gbp5*, and *Tifa*), Neu-2 (mainly expressing *Myadm*, *Cd3*00ld, and *Ly6g*), and Neu-3 (mainly expressing *Lta4h*, *Sort1*, and *Rgs18*) (Fig. 3A, B, and D, and Fig. S3A). Neu-2 was the major subcluster of neutrophils and accounted for up to 71.54% of liver neutrophils in healthy mice (Fig. 3C). Neu-1 was the major subcluster (60.61%) of neutrophils at the early stage of CLP (6 h), whereas the neutrophils in Neu-3 constituted the major subcluster (48.16%) of neutrophils at 24 h after CLP. Impressively, the proportion of Neu-3 in the liver exhibited a continuous increase in a time-dependent manner during sepsis, which were verified via immunofluorescence staining of Ly6G, Lta4h, and Sort1 in healthy and sepsis mice (Fig. 3G). In addition, our results indicate that the changes in the number of total neutrophils in different groups were consistent with the scRNA-seq result (Fig. S3B).

Further analysis revealed that neutrophil subclusters exhibited different transcriptional characteristics (Fig. 3E and F). Both Neu-1 and Neu-3 were inflammatory neutrophils that bore a pro-inflammatory gene signature. Neu-1 showed a high expression of pro-inflammatory cytokines (*Il1b*, *Tnf*, and *Mif*), neutrophil-attracting chemokines (*Cxcl1*, *Cxcl2*, and *Cxcl3*), and adhesion molecule (*Icam1*) (Fig. 3E). Notably, Neu-1 also exhibited a high expression of immune regulatory genes (*Cd274* and *Socs3*) (Fig. 3F). Neu-3 showed a hyperactivated phenotype with a high expression of calgranulins (*S100a9* and *S100a8*), neutrophil activation markers (*Mpo*, *Ltf*, and *Cd69*), neutrophil infiltration-related genes (*Cd47*, *Spn/Cd4*3, *Mmp8*, and *Mmp25*), and pro-inflammatory cytokines (*Il-6*) (Fig. 3E and F). In addition, Neu-3 showed apoptotic properties via the upregulation of the pro-apoptosis-related genes (*Fadd*, *Casp6*, *Bad*, and *Bclaf1*) (Fig. 3F). Collectively, it is suspected that Neu-1 responded to the microbial invasion in the liver, and became activated to eliminate microbial organisms, while concomitantly releasing inflammatory cytokines and chemokines to recruit more immune cells, including Neu-3, to the liver. Consequently, a significant number of activated Neu-3 with robust pathogen removal capability could have infiltrated the liver and eliminated the invading microbial organisms via phagocytosis and the release of cytotoxic antimicrobial molecules (reactive oxygen species and lactoferrin), ultimately leading to liver injury.

KEGG pathway analyses revealed that the genes that were upregulated in Neu-1 belonged to the NOD-like receptor, TNF/NF-κB, C-type lectin receptor, and Toll-like receptor signalling pathways (Fig. 3H). Meanwhile, the genes that were upregulated in Neu-3 belonged to the leucocyte transendothelial migration, endocytosis, and phagosome (Fig. 3H).

SCENIC analysis was performed to assess which TFs were responsible for the differences in gene expression between different cell clusters. *Hif1a*, *Atf4*, *Atf6*, *Irf1*, *Nfkb1*, and *Irf5* were identified as candidate TFs underlying the gene signature of Neu-1 (Fig. 3I), whereas *Xbp1*, *Fosl1*, and *Ltf* were the candidate TFs underlying the differential gene expression in Neu-3.

### scRNA-seq revealed heterogeneity of Kupffer cells in liver during sepsis

**Fig. 4:**
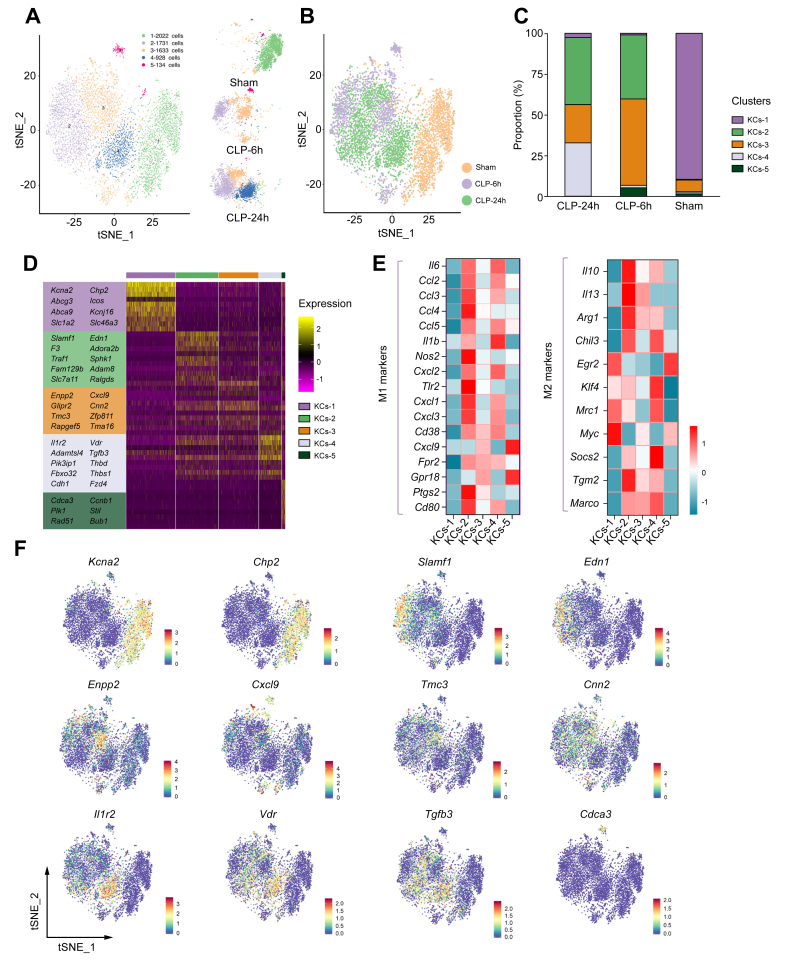
Hepatic Kupffer cell dynamics and functionality in healthy and septic livers. (A) Subclustering of Kupffer cells in healthy and septic livers. (B) UMAP plot of Kupffer cells across the indicated conditions. (C) The proportion of Kupffer cell subclusters in each sample. (D) Heatmap plots demonstrating expression of specified genes among Kupffer cell subclusters. (E) Heatmap showing gene expression by the indicated Kupffer cell subclusters. (F) UMAP plot showing subcluster-specific marker genes of Kupffer cells. CLP, caecal ligation and puncture; CLP-24 h, 24 h after CLP; CLP-6h, 6 h after CLP; KCs-1 to KCs-4, Kupffer cell subclusters 1 to 4; tSNE, t-distributed stochastic neighbour embedding; UMAP, uniform manifold approximation and projection.

Kupffer cells represent the major fraction of liver macrophages^37^ and play a pivotal role in maintaining homoeostasis of the liver as well as in contributing to the progression of acute liver dysfunction.^38^ In this study, Kupffer cells were grouped into five clusters (Fig. 4A, B, D, and F), which were annotated as KCs-1 (enriched in the expression of *Kcna2*, *Abcg3*, and *Slc1a2*), KCs-2 (enriched in the expression of *Slamf1*, *Edn1*, *Adora2b*, and *Adam8*), KCs-3 (enriched in the expression of *Enpp2*, *Cxcl9*, *Tmc3*, and *Cnn2*), KCs-4 (enriched in the expression of *Il1r2*, *Vdr*, *Tgfb3*, and *Cdh1*), and KCs-5 (enriched in the expression of *Cdca3*, *Stil*, and *Rad51*). KCs-1 constituted up to 89.48% of Kupffer cells under healthy conditions, whereas KCs-3 constituted only 7.29% of the total Kupffer cells (Fig. 4C). During the early stage of sepsis (6 h), the proportions of KCs-2 and KCs-3 were increased and accounted for 92.21% of all Kupffer cells. As liver injury progressed in severity, the proportion of KCs-2 increased slightly (39.20 to 40.93%), and the proportion of KCs-3 decreased considerably (53.01 to 23.61%) at 24 h after CLP. Importantly, KCs-4 increased from less than 2% of total Kupffer cells in the sham and CLP-6h groups to 32.57% in the CLP-24 h group. Based on the dynamic changes in the proportion of Kupffer cell subclusters, it is suspected that KCs-2, KCs-3, and KCs-4 play important roles in the development of sepsis-induced acute liver dysfunction. In addition, total Kupffer cells were detected using F4/80 immunofluorescence staining. Our results indicate that the changes in the number of F4/80 positive cells in different groups were consistent with the scRNA-seq result (Fig. S4).

Further analysis revealed that KCs-1, KCs-2, KCs-3, and KCs-4 exhibited different expression profiles of inflammation-related genes (Fig. 4E). KCs-2 showed not only a high expression of pro-inflammatory genes/M1 markers (*e.g. Il-6*, *Ccl2*, *Ccl3*, *Ccl4*, *Nos2*, *Cxcl1*, *Cxcl2*, *Cxcl3*, *Tlr2*, *Ptgs2*, *Fpr2*, *Cd80*, and *Cd38*) but also a high expression of several feedback inhibitors of activation (*Il10*, *Il13*, *Arg1*, and *Tgm2*). KCs-3 also exhibited a high expression of pro-inflammatory genes, specifically *Cxcl9*, *Fpr2*, and *Gpr18.* Similarly, KCs-4 showed a relatively high expression of pro-inflammatory genes (*Il-6*, *Ccl2*, *Ccl3*, *Ccl5*, *Il-1b*, *Cd38*, *Cxcl2*, and *Fpr2*), as well as genes associated with M2-like Kupffer cells (*Il10*, *Mrc1*, *Socs2*,^39^ *Arg1*, *Klf4*,^40^ and *Chil3*^41^). In contrast, KCs-1 exhibited a very low expression of pro-inflammatory genes and a high expression level in genes associated with M2-like Kupffer cells (*Myc* and *Egr2*). These results indicated that KCs-2, KCs-3, and KCs-4 were activated and played a vital role in releasing cytokines and chemokines, which most likely activated and recruited circulating macrophages and neutrophils to liver tissue, subsequently leading to liver injury.

### Cell-to-cell communication in CLP-induced liver dysfunction: receptor–ligand analysis

**Fig. 5:**
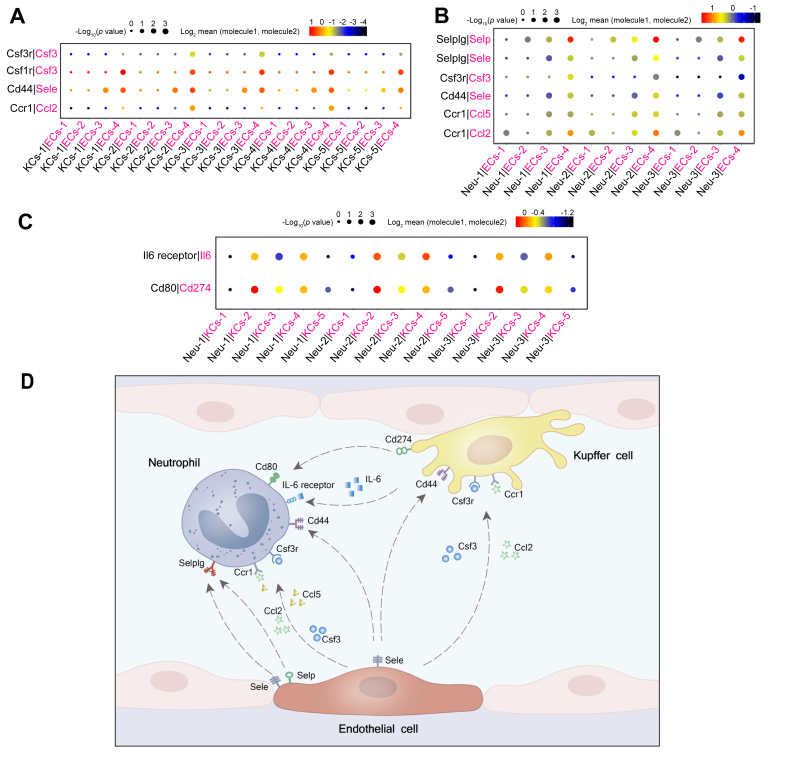
Molecular interactions of neutrophils, endothelial cells, and Kupffer cells. (A) Interactions between four endothelial cell subclusters and five Kupffer cell subclusters. (B) Interactions between three neutrophil subclusters and four endothelial cell subclusters. (C) Interactions between three neutrophil subclusters and four Kupffer cell subclusters. Dot sizes and colours represent logarithmic-transformed *p* values and mean expression of interacting molecules in corresponding cells. (D) Predicted main regulatory network among endothelial cells, neutrophils, and Kupffer cells during sepsis. ECs-1 to ECs-4, endothelial cell subclusters 1 to 4; KCs-1 to KCs-5, Kupffer cell subclusters 1 to 5; Neu-1 to Neu-3, neutrophil subclusters 1 to 3.

Elucidating the explicit interaction among liver cells during sepsis will shed light on the pathogenesis of sepsis-induced acute liver dysfunction. The ligand–receptor pairs among the major cell types are shown in Fig. S5. Notably, the Kupffer cells showed the most interactions with other cell types during sepsis, in particular with endothelial cells and neutrophils, at 24 h after sepsis. To further explore the detailed cellular communication during CLP-induced liver injury, the intercellular interactions within heterogeneous populations of neutrophils, Kupffer cells, and endothelial cells were analysed. As shown in Fig. 5A, ECs-4, the major subcluster of endothelial cells at 24 h after CLP, showed enhanced interactions with Kupffer cells via the Csf3r/Csf3, Csf1r/Csf3, CD44/Sele, and Ccr1/Ccl2 axes (Fig. 5A). Furthermore, endothelial cell–neutrophil interactions are crucial for neutrophil infiltration to the liver.^13^ Notably, ECs-4, the major subcluster of endothelial cells at 24 h after CLP, expressed relatively high levels of chemokines (*Ccl2* and *Ccl5*), cytokines (*Csf3*/*G-CSF*), and adhesion molecules (*Sele* and *Selp*), whereas the corresponding receptors were widely expressed in Neu-3, suggesting that functional interactions between Neu-3 and ECs-4 may play significant roles in enhancing neutrophil infiltration into liver tissues (Fig. 5B). In addition, the enhanced interactions between Kupffer cells (KCs-2, KCs-3, and KCs-4) and neutrophils, such as Il6 receptor/Il6 and Cd80/Cd274, were the most noticeable (Fig. 5C).

Our results found that the activated endothelial cells secrete colony-stimulating factors (Csf3) and chemokines (Ccl2) to interact with immune cells including Kupffer cells and neutrophils, and facilitate the immune cell adhesion via elevating the adhesion molecule expression (Sele and Selp) during sepsis (Fig. 5D), which can provide precise drug targets for early treatment of sepsis.

### ATF4 inhibition alleviated CLP-induced acute liver dysfunction and prolonged the survival of septic mice

In the present study, the TFs underlying the differential gene expression in endothelial cells, Kupffer cells, neutrophils, and monocytes/monocyte-derived macrophages during the sepsis-induced acute liver dysfunction were identified (Fig. S6A–D), and the representative results were verified via immunofluorescence. As shown in Fig. S5E and F, a rapid increase of ATF4 and NF-κB1 expression in endothelial cells were observed 6 h after CLP when compared with that of healthy controls. Subsequently, the ATF4 and NF-κB1 expression declined at 24 h after CLP compared with that at 6 h after CLP. In addition, the Fosl1 expression in Kupffer cells increased with pathological progression (Fig. S6G).

Among these TFs, ATF4 was identified as the hallmark TF in endothelial cells, Kupffer cells, neutrophils, and monocytes/monocyte-derived macrophages at 6 h after sepsis (Fig. S6A–D). These results suggested that ATF4 activated the endothelial and immune cells associated with the hyperinflammatory response, which contributed to the pathogenesis of CLP-induced acute liver dysfunction in the early stage of sepsis; thus, we hypothesised that the inhibition of ATF4 would attenuate the CLP-induced acute liver dysfunction.

**Fig. 6:**
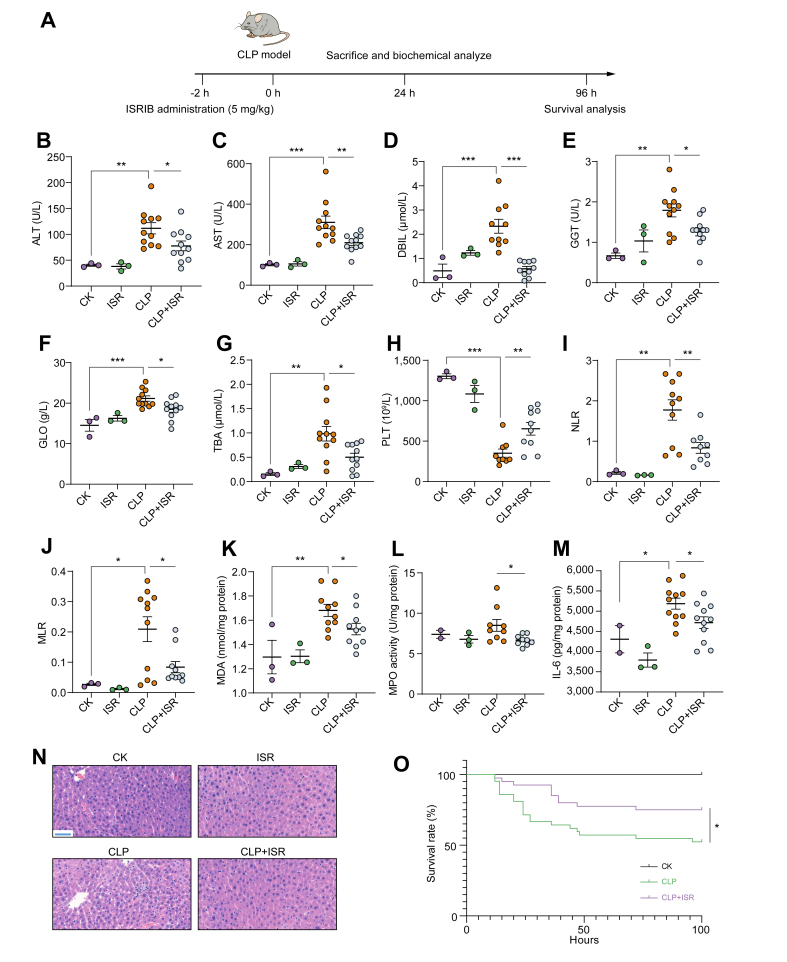
Therapeutic effects of ATF4 inhibition on sepsis-induced acute liver dysfunction and survival in CLP-induced sepsis model. (A) Experimental timeline of ATF4 inhibition in CLP-induced sepsis model (*in vivo*). The levels of (B) ALT, (C) AST, (D) DBIL, (E) GGT, (F) GLO, and (G) TBA in plasma 24 h after CLP. The (H) PLT in blood, (I) NLR, and (J) MLR. The (K) MDA content, (L) MPO activity, and (M) IL-6 in liver 24 h after CLP. (N) Representative images show the effect of ISRIB administration on histological injury (scale bar, 50 μm). (O) Survival rates of sepsis mice after 96 h following ISRIB administration. ∗*p* <0.05; ∗∗*p* <0.01; ∗∗∗*p* <0.001. Statistical differences between groups were assessed using one-way ANOVA for (B)–(G). Statistical differences between groups were assessed using a *t* test for (K)–(M). Statistical differences between groups were analysed using the log-rank test for (O). ALT, alanine aminotransferase; AST, aspartate aminotransferase; ATF4, activating transcription factor 4; CLP, caecal ligation and puncture; DBIL, direct bilirubin; GGT, glutamyl-transpeptidase; GLO, globulin; ISR, ISRIB; ISRIB, integrated stress response inhibitor; MDA, malondialdehyde; MLR, monocyte-to-lymphocyte ratio; MPO, myeloperoxidase; NLR, neutrophil-to-lymphocyte ratio; PLT, platelet counts; TBA, total bile acid.

ISRIB, a specific small-molecule inhibitor, can suppress the upregulation of ATF4 and decrease nuclear translocation of ATF4.^42^ As shown in Fig. 6A and B, the plasma ALT and AST activities were used as biomarkers of liver dysfunction. CLP significantly elevated the plasma AST and ALT activities compared with those in the normal controls, which indicated liver dysfunction in the CLP group. ISRIB administration significantly reduced the other liver dysfunction biomarkers, including direct bilirubin, glutamyl-transpeptidase, globulin, total bile acid, and albumin-to-globulin ratio, in plasma compared with those of the CLP group (Fig. 6D–G and Fig. S6H). Notably, ISRIB administration alone showed no significant differences in the liver dysfunction biomarkers compared with those in the normal controls (*p* >0.05), which suggested that ISRIB exerted no acute toxicity at the administered dosage.

Elevated neutrophil-to-lymphocyte ratio and monocyte-to-lymphocyte ratio have been proposed as indicators of a systemic inflammatory response.^43^^,^^44^ Recently, research has exhibited that severe cases of COVID-19 tended to have a higher neutrophil-to-lymphocyte ratio.^43^^,^^44^ Blood analysis demonstrated that CLP significantly reduced the platelet count and increased the neutrophil-to-lymphocyte ratio and monocyte-to-lymphocyte ratio, which were markedly reversed by ISRIB treatment (Fig. 6H–J). These results indicated that ISRIB treatment could decrease the systemic inflammatory response during the early stage of sepsis. In addition, the ISRIB treatment significantly elevated the number of lymphocytes and lymphocyte percentage compared with that of the CLP group (Fig. S6J and I), which suggests that ISRIB treatment promotes immune cell survival and maintains immune function during sepsis.

Hepatic lipid peroxidation and neutrophil infiltration are detected by quantifying the MDA content and MPO activity.^13^^,^^45^ As shown in Fig. 6K, a significantly higher hepatic MDA content was observed in septic model mice compared with that in the normal control, and the mice administered with ISRIB exhibited a significantly decreased MDA content compared with that in the sepsis model mice. ISRIB administration significantly inhibited neutrophil infiltration in treated mice compared with that in untreated sepsis model mice (Fig. 6L). In comparison with that in the normal control, significantly higher hepatic IL-6 level was indicated in the sepsis model mice treated with vehicle. ISRIB treatment significantly diminished the hepatic IL-6 level compared with that in the sepsis control (Fig. 6M).

As shown in Fig. 6N and Fig. S6K, we observed a marked liver injury including cell swelling, inflammatory cell infiltration, and tissue architecture disruption in sepsis model mice, whereas ISRIB administration exhibited a significant improvement in the pathological injury. To further explore the therapeutic potential of ISRIB, the effect of ISRIB administration on the survival of CLP-induced septic mice was examined. The CLP induced the death in mice, and a single-dose administration of ISRIB significantly improved the survival of CLP mice from 52 to 75% (*p* <0.05; Fig. 6O).

## Discussion

Liver tissue is composed of multiple nonparenchymal cell lineages including neutrophils, endothelial cells, and Kupffer cells, all of which are essential contributors to uncontrolled local inflammation during sepsis exposure, causing intractable liver injury. The characteristics of gene heterogeneity differentiate these major cell types and define multiple subpopulations with distinct functions. Therefore, resolving the dynamic changes of the transcriptome at the single-cell level during disease progression is critical to understanding the intrinsic mechanisms of sepsis-induced acute liver dysfunction, which is of great significance for improving disease diagnosis and intervention. In the current study, scRNA-seq was used to characterise the dynamic cellular and molecular signatures along the disease course, and the key findings were validated in a CLP-induced sepsis model.

This study provides new insights into the role of endothelial cell states in the pathogenesis of sepsis-induced acute liver dysfunction. The number of hepatic endothelial cells was observed to be decreasing in a time-dependent manner during the progression of sepsis, which is consistent with a recent study in an endotoxemia model.^46^ We speculated that the weak cellular adhesion and high apoptosis revealed by scRNA-seq might be the main cause of the sharp reduction in the number of endothelial cells, which then triggers interstitial oedema and consecutively worsens liver hypoperfusion, accelerating liver injury.^47^ In addition, pieces of clinical evidence also indicate that endothelial damage occurred during the pathogenesis of sepsis and COVID-19 infection,^48^^,^^49^ leading to an increased circulating endothelial cell, endothelium barrier dysfunction,^50^ and subsequently organ injury including acute liver injury.^51^ These experimental and clinical results suggest the importance of endothelium protection in early sepsis.

Endothelial cells could undergo dynamic phenotypic switching when exposed to various environments.^51^^,^^52^ We found that hepatic endothelial cells could be reprogrammed into cells displaying pro-inflammatory phenotype during sepsis, as ECs-3 and ECs-4 were observed to be in different pro-inflammatory states reflected by their inflammation-related gene expression profiles. Particularly, this result indicated that ECs-3 may play an important role in the adaptive immune response to infection as antigen-presenting cells, as reported in other studies as well.^53^^,^^54^ NOD1 is an important and well-characterised member of the NOD-like receptor family, which was demonstrated to enhance antigen-presenting ability of liver sinusoidal endothelial cells during viral infection.^55^ We also found *Nod1* to be highly expressed in ECs-3. Thus, NOD1 is likely to be involved in the antigen-presenting activity of ECs-3 during sepsis. In addition, ECs-4 was elicited to be in an endothelial activation/dysfunction state owing to a high expression of endothelial activation/dysfunction markers, including Vcam-1, Icam-1, Selp, Sele, Serpine1, and Fgf23.^18^^,^^21^^,^^22^^,^^56^ The activated ECs-4 could interact with neutrophils and exacerbate sepsis-induced acute liver dysfunction by cell-to-cell communication.^13^ We found that the Csf3r/Csf3 axis was only presented in the interactions between neutrophils and activated ECs-4, suggesting that blocking the Csf3r/Csf3 axis may be a potential therapeutic avenue in treating sepsis-induced acute liver dysfunction, but this still requires further research.

Kupffer cells are a critical component of the mononuclear phagocytic system and are central to both the hepatic and systemic response to pathogens.^57^ Previous studies have indicated that the polarisation of Kupffer cells is recognised as a critical mediator of liver injury.^3^^,^^58^ Traditionally, Kupffer cells are polarised into distinct phenotypes, M1 (inflammatory) and M2 (anti-inflammatory), depending on the local microenvironment.^58^ In this study, beyond the quiescent KCs-1, the inflammatory Kupffer cells including KCs-2, KCs-3, and KCs-4 were also identified, and the expression of M1 and M2 Kupffer cell markers were subsequently determined in all five Kupffer cell subpopulations. We found that all the subpopulations showed dual expression of pro-inflammatory genes and anti-inflammatory genes. Specifically, although IL-10, Arg1, and Chil3 were reported as classical M2 Kupffer cell markers,^59^ the inflammatory Kupffer cell subpopulations, namely KCs-2, KCs-3, and KCs-4, with their high expression of pro-inflammatory genes, also exhibited a high level of expression of IL-10, Arg1, and Chil3. Similar phenomena have also been reported in other studies.^60^^,^^61^ Altogether, these results indicated that Kupffer cells are highly plastic and could undergo a broad spectrum of transcriptomic activation states under a complex hepatic microenvironment and cannot be simply classified into either M1 or M2 polarisation. A more comprehensive classification system based on recent research is required to describe the activation states of Kupffer cells *in vivo.*

As the solution injected into the abdominal cavity could flow out though the wound, we do not have the data to evaluate the therapeutic effect if ISRIB was to be given after CLP. Our results indicate that the ATF-4 activation occurs at 6 h after CLP and returns to normal at 24 h after CLP. We speculate that ISRIB exhibits therapeutic effect if ISRIB is given early (<6 h) and that the therapeutic effect might decrease with a prolonged administration time.

In summary, our study presents the dynamic transcriptomic landscape of major nonparenchymal cells at single-cell resolution, in which we identified the significant alterations and heterogeneity of hepatic nonparenchymal cell subsets during sepsis. Importantly, we identified the endothelial cell and neutrophil subsets that are associated with acute liver dysfunction during sepsis progression and explored the therapeutic effect of ATF4 inhibition. Overall, these results uncovered potential mechanisms and promising therapeutic targets for the prevention and treatment of sepsis-induced acute liver dysfunction and other liver-related diseases.

## Financial support

This work was supported by the 10.13039/501100001809National Natural Science Foundation of China (82170229) and Foundation Strengthening Program Technology Fund Project (2019-JCJQ-JJ-164).

## Authors’ contributions

Designed the study: CG, YY, ZH. Performed the experiments and collected the data: CG, RC, XY. Analysed the data: CG, XY, RC, WY, YR, WQ, YG, LM, ZX, YS, ZJ. Prepared the manuscript: LM, CG, RC, XY.

## Data availability statement

The data that support the findings of this study are available from the corresponding authors upon reasonable request.

## Conflicts of interest

The authors declare no conflict of interest.

Please refer to the accompanying ICMJE disclosure forms for further details.
