# Supplementary material for: Time-resolved single-cell transcriptomics reveals the landscape and dynamics of hepatic cells in sepsis-induced acute liver dysfunction
Source: JHEP Rep. 2023 Mar 1;5(6):100718. doi: 10.1016/j.jhepr.2023.100718 (PMC10130477; doi:10.1016/j.jhepr.2023.100718)
Supplement: Multimedia component 1 [file mmc1.pdf]

Time-resolved single-cell transcriptomics reveals the landscape and dynamics of hepatic cells in sepsis-induced acute liver dysfunction

Gan Chen, Chao Ren, Yao Xiao, Yujing Wang, Renqi Yao, Quan Wang, Guoxing You, Mingzi Lu, Shaoduo Yan, Xiaoyong Zhang, Jun Zhang, Yongming Yao, Hong Zhou

Table of contents:

Fig. S1

Fig. S2

Fig. S3

Fig. S4

Fig. S5

Fig. S6

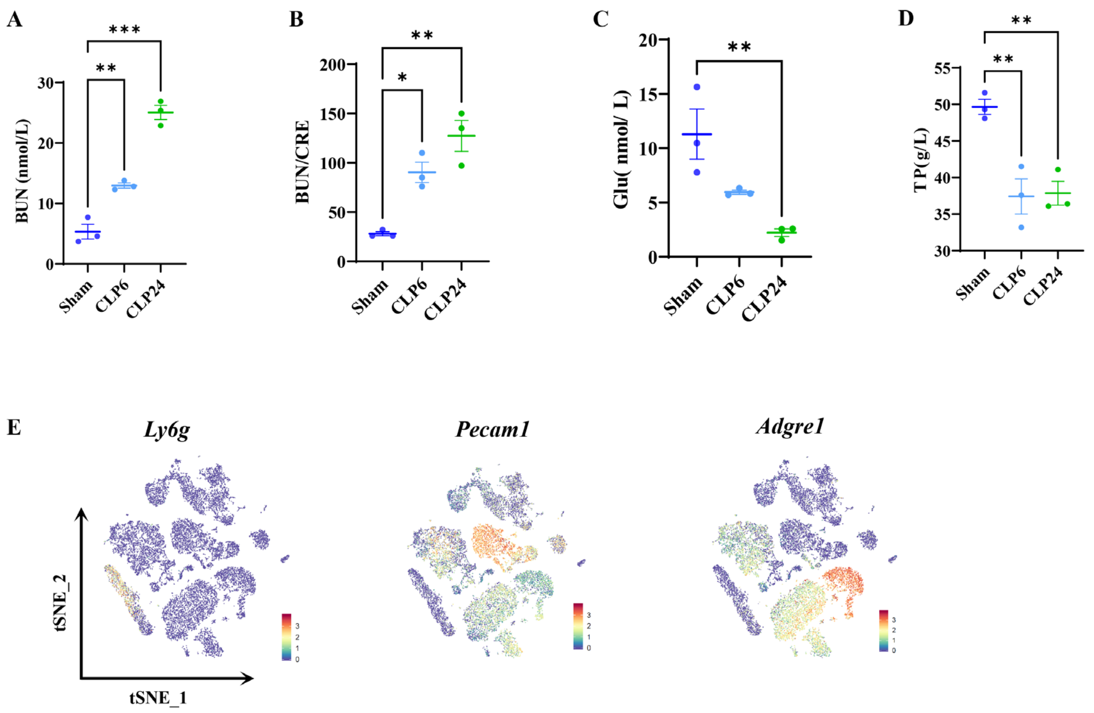
A
B
C
D
E
BUN (nmol/L)
BUN/CRE
Glu(nmol/ L)
TP(g/L)
Sham
CLP6
CLP24
***
**
*
Ly6g
Pecam1
Adgre1
tSNE_2
tSNE_1

F

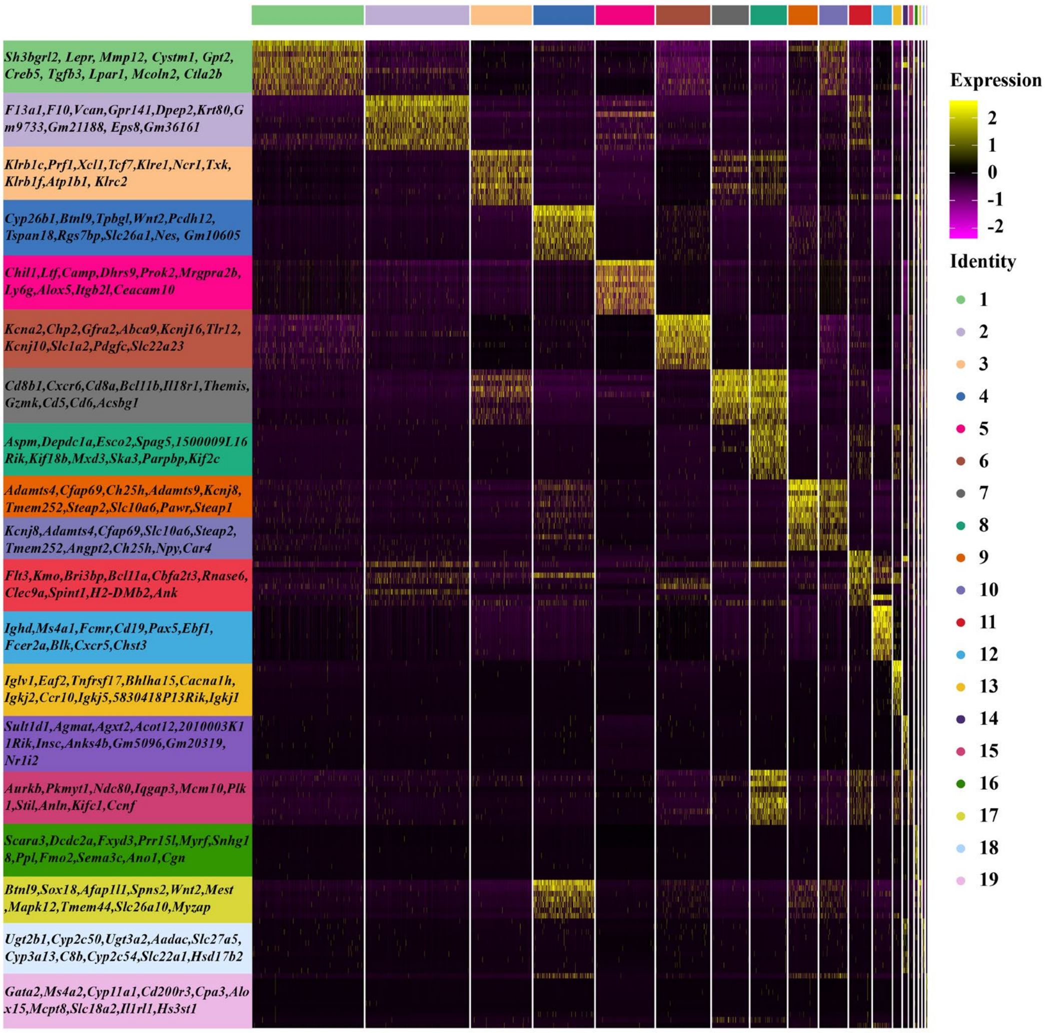

Fig. S1. Biochemical indices in plasma. The levels of blood urea nitrogen (BUN) (A), blood urea nitrogen/creatinine (BUN/CRE) (B), glucose (Glu) (C), total protein (TP) (D) in plasma. (E) The cell markers indicating group identities. (F) Heatmap plots demonstrating expression of specified genes of 19 cell clusters. *$p < 0.05$; **$p < 0.01$; ***$p < 0.001$. Statistical differences between groups were performed by a one-way ANOVA for Fig. S1 A, B, C, and D.

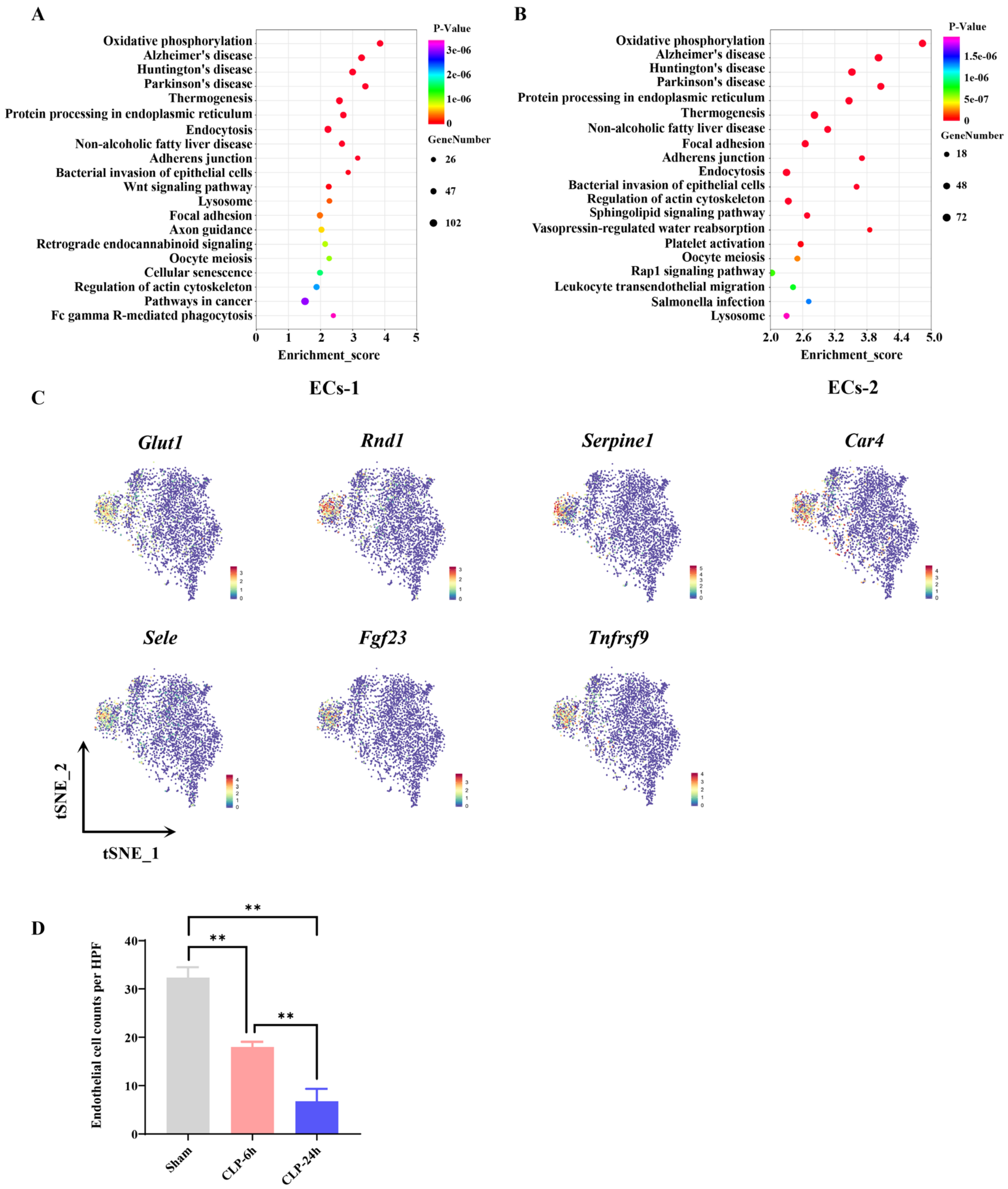

Fig. S2. The enriched pathway of associated with endothelial cell subclusters 1 (A) and 2 (B). Colors indicate significance of enrichment and circle sizes indicate number of genes falling into respective categories. (C) The markers indicating group identities. (D) The average number of neutrophils per high-power field (HPF). **p < 0.01. Statistical differences between groups were performed by a one-way ANOVA.

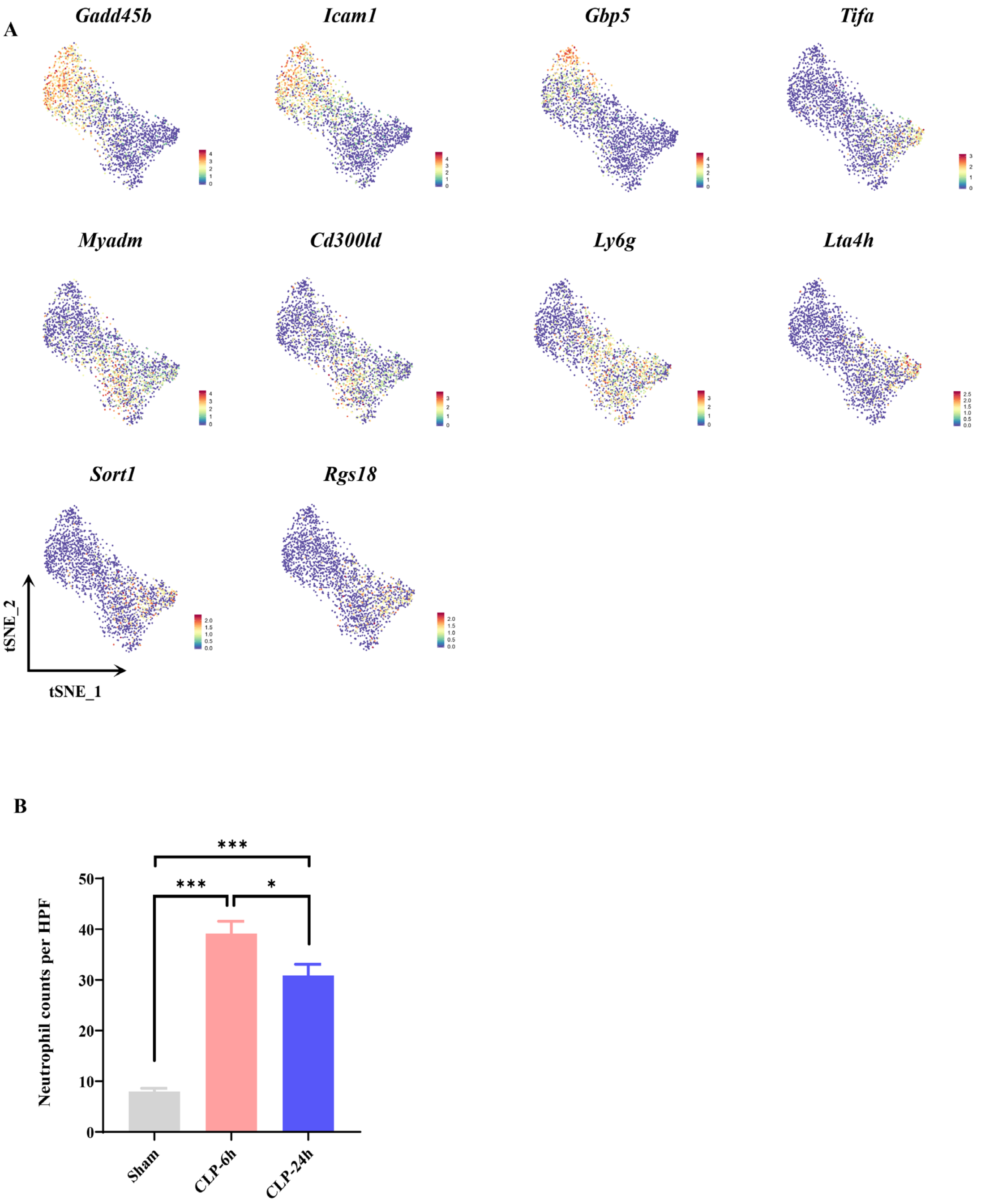

Fig. S3. (A) The markers indicating group identities. (B) The average number of neutrophils (Ly6G positive cells) per high-power field (HPF). *$p < 0.05$; ***$p < 0.001$. Statistical differences between groups were performed by a one-way ANOVA.

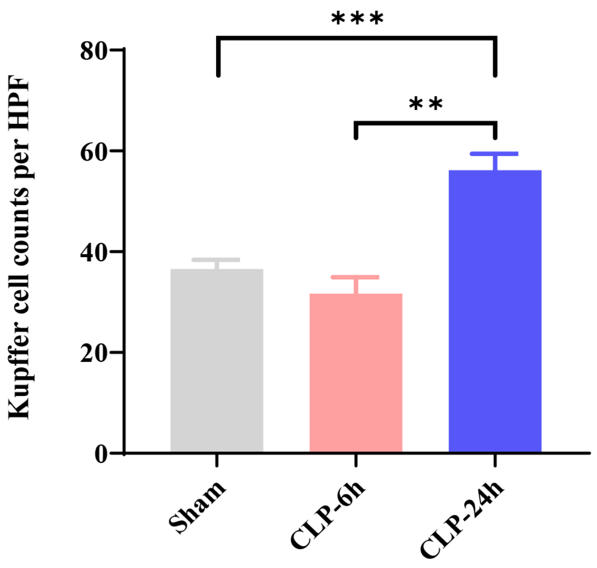

Fig. S4. The average number of Kupffer cells per high-power field (HPF). ***p* < 0.01; ****p* < 0.001. Statistical differences between groups were performed by a one-way ANOVA.

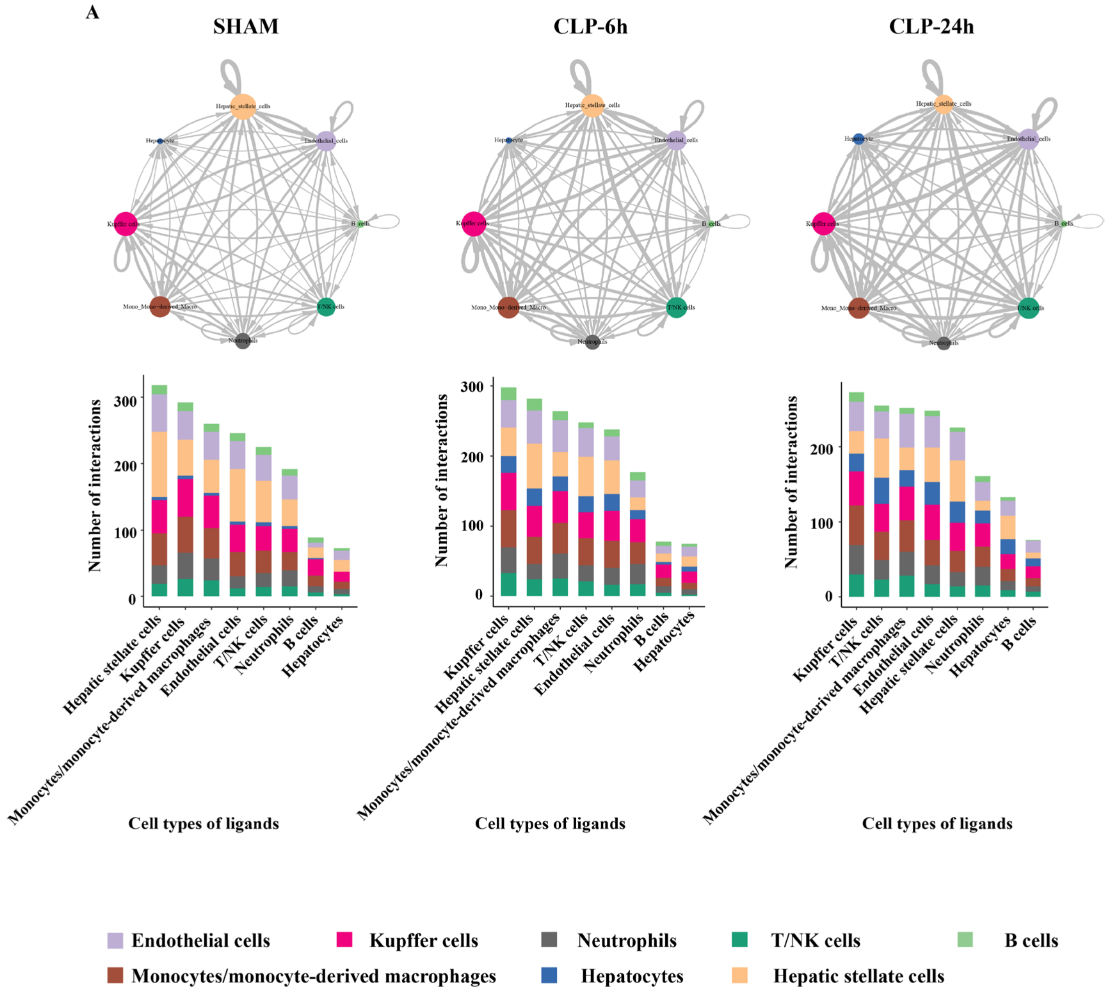

Fig. S5. The cell-cell interaction network and histogram plot shows interaction frequencies between different cell types across the indicated conditions. Colorful nodes represent cell types, and their sizes indicate interaction numbers.

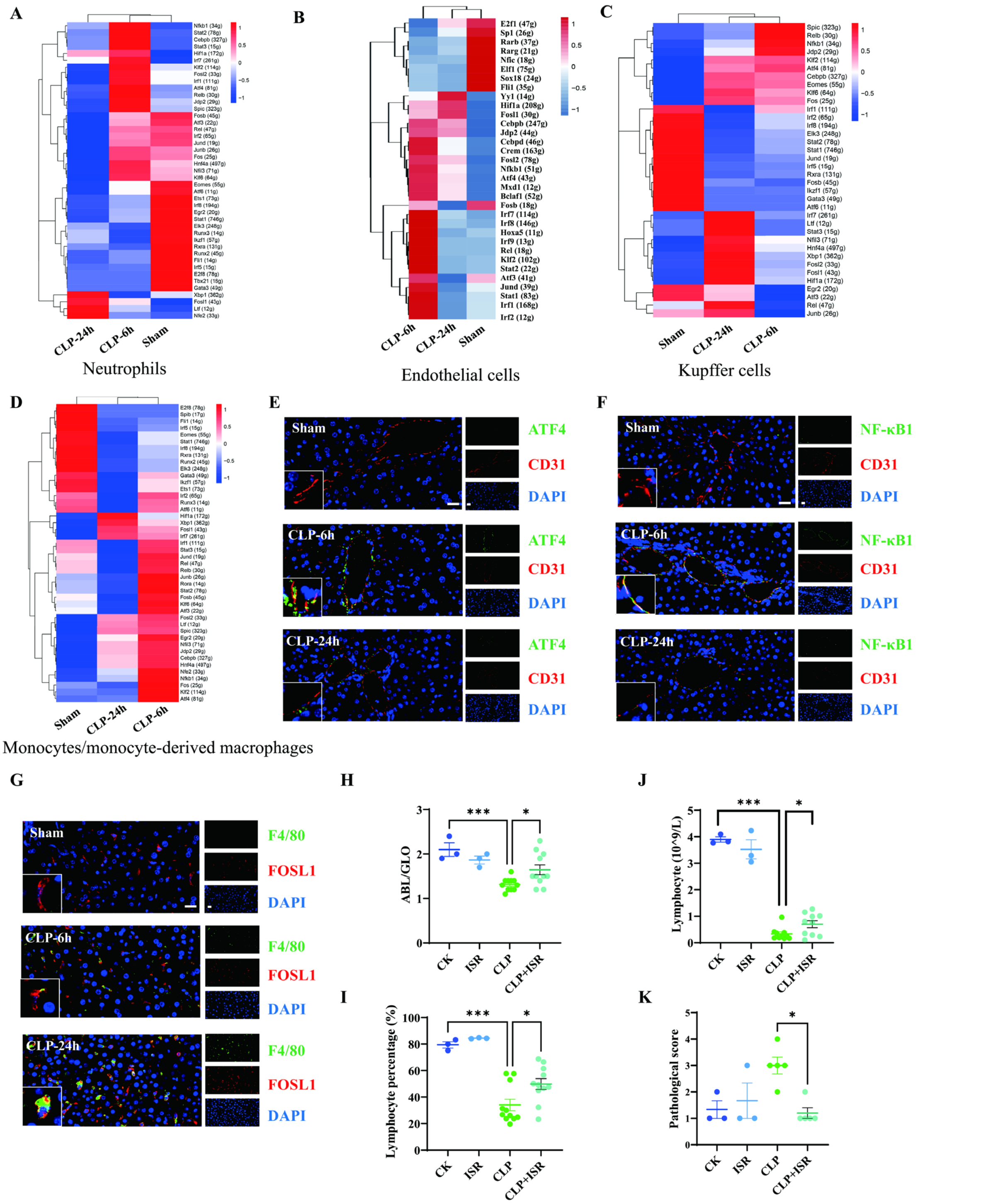

Fig. S6. (A) The specific regulons of each condition for neutrophils revealed by SCENIC analysis. (B) The specific regulons of each condition for endothelial cells revealed by SCENIC analysis. (C) The specific regulons of each condition for Kupffer cells revealed by SCENIC analysis. (D) The specific regulons of each condition for monocytes/monocyte-derived macrophages revealed by SCENIC analysis. Immunofluorescence staining results showing the protein expression of ATF4 (E) and

NF-κB1 (F) in endothelial cells of healthy and septic mouse livers. Scale bars, 20 μm. (G) Immunofluorescence staining results showing the protein expression of FOSL1 in Kuppfer cells of healthy and septic mouse livers. Scale bars, 20 μm. (H) The levels of albumin (ALB)/globulin (GLB) ratio in plasma 24 h after CLP. The levels of lymphocyte percentage (I) and count (J) in blood 24 h after CLP. K. Histologic liver injury scores. ISR: ISRIB, *$p < 0.05$; * * *$p < 0.001$. Statistical differences between groups were performed by a one-way ANOVA for Fig. S6 H, J, and I. Statistical differences between groups were performed by t-test for Fig. 6K.
