# Supplementary material for: Time-resolved single-cell transcriptomics reveals the landscape and dynamics of hepatic cells in sepsis-induced acute liver dysfunction
Source: JHEP Rep. 2023 Mar 1;5(6):100718. doi: 10.1016/j.jhepr.2023.100718 (PMC10130477; doi:10.1016/j.jhepr.2023.100718)
Supplement: Multimedia component 2 [file mmc2.docx]

**JHEP Reports**

**CTAT methods**

Tables for a “Complete, Transparent, Accurate and Timely account” (CTAT) are now mandatory for all revised submissions. The aim is to enhance the reproducibility of methods.

- Only include the parts relevant to your study
- Refer to the CTAT in the main text as ‘Supplementary CTAT Table’
- Do not add subheadings
- Add as many rows as needed to include all information
- Only include one item per row

**If the CTAT form is not relevant to your study, please outline the reasons why:**

|  |
| --- |

- 1. **Antibodies**

| **Name** | **Citation** | **Supplier** | **Cat no.** | **Clone no.** |
| --- | --- | --- | --- | --- |
| anti-CD31 primary antibody |  | Proteintech | 17617-1-AP |  |
| anti-Glut1 primary antibody |  | Proteintech | 21829-1-AP |  |
| anti-Sele primary antibody |  | Proteintech | 20894-1-AP |  |
| anti-Ly6G primary antibody |  | Servicebio | GB11229 |  |
| anti-Lta4h primary antibody |  | Proteintech | 13662-1-AP |  |
| anti-Sort1 primary antibody |  | Proteintech | 12369-1-AP |  |
| anti-ATF4 primary antibody |  | ABclonal | A18687 |  |
| anti-Fosl1 primary antibody |  | ABclonal | A5372 |  |
| anti-NF-κB1 primary antibody |  | ABclonal | A6667 |  |
| anti-F4/80 primary antibody |  | Servicebio | GB113373 |  |

- 1. **Organisms**

| **Name** | **Citation** | **Supplier** | **Strain** | **Sex** | **Age** | **Overall n number** |
| --- | --- | --- | --- | --- | --- | --- |
| Mice |  | Beijing Vital River Laboratories | C57BL6/J | male | 7–8 weeks |  |

- 1. **Software**

| **Software name** | **Manufacturer** | **Version** |
| --- | --- | --- |
| Prism 8 | GraphPad Software | 8.0.2 |
| BD Rhapsody Analysis Pipeline | BD Biosciences | 1.9 |
| R package Seurat |  | 3.1.1 |
| RcisTarget and GRNboost |  | 1.1.2.2 |
| CellChat R package |  | 1.1.3 |
| GRNboost |  | 1.1.2.2 |
| AUCell |  | 1.4.1 |
| RcisTarget |  | 1.2.1 |

- 1. **Other (*e.g*. drugs, proteins, vectors etc.)**

| ISRIB | Beyotime Biotechnology | SC4332 |
| --- | --- | --- |
|  |  |  |

- 1. **Please provide the details of the corresponding methods author for the manuscript:**

| **Gan Chen, Yao Xiao, Chao Ren, Yujing Wang, Quan Wang, Guoxing You** |
| --- |
