# Supplementary material for: Time-resolved single-cell transcriptomics reveals the landscape and dynamics of hepatic cells in sepsis-induced acute liver dysfunction
Source: JHEP Rep. 2023 Mar 1;5(6):100718. doi: 10.1016/j.jhepr.2023.100718 (PMC10130477; doi:10.1016/j.jhepr.2023.100718)
Supplement: Multimedia component 4 [file mmc4.pdf]

Time-resolved single-cell transcriptomics reveals the landscape and dynamics of hepatic cells in sepsis-induced acute liver dysfunction

Gan Chen,[1,*,†] **Chao Ren**,[2,3,†] **Yao Xiao**,[1,†] Yujing Wang,[1] Renqi Yao,[2] Quan Wang,[1] Guoxing You,[1] Mingzi Lu,[4] Shaoduo Yan,[1] Xiaoyong Zhang,[1] Jun Zhang,[1] Yongming Yao,[2,*] Hong Zhou[1,*]

[1]Institute of Health Service and Transfusion Medicine, Academy of Military Medical Sciences, Beijing, China; [2]Translational Medicine Research Center, Fourth Medical Center and Medical Innovation Research Division of the Chinese PLA General Hospital, Beijing, China; [3]Department of Pulmonary and Critical Care Medicine, Beijing Chaoyang Hospital, Capital Medical University, Beijing, China; [4]Beijing Science and Technology Innovation Research Center, Beijing, China

JHEP Reports **2023.** https://doi.org/10.1016/j.jhepr.2023.100718

Background & Aims: Sepsis-induced acute liver dysfunction often occurs early in sepsis and can exacerbate the pathology by triggering multiple organ dysfunction and increasing lethality. Nevertheless, our understanding of the cellular heterogeneity and dynamic regulation of major nonparenchymal cell lineages remains unclear.
Methods: Here, single-cell RNA sequencing was used to profile multiple nonparenchymal cell subsets and dissect their crosstalk during sepsis-induced acute liver dysfunction in a clinically relevant polymicrobial sepsis model. The transcriptomes of major liver nonparenchymal cells from control and sepsis mice were analysed. The alterations in the endothelial cell and neutrophil subsets that were closely associated with acute liver dysfunction were validated using multiplex immunofluorescence staining. In addition, the therapeutic efficacy of inhibiting activating transcription factor 4 (ATF4) in sepsis and sepsis-induced acute liver dysfunction was explored.
Results: Our results present the dynamic transcriptomic landscape of major nonparenchymal cells at single-cell resolution. We observed significant alterations and heterogeneity in major hepatic nonparenchymal cell subsets during sepsis. Importantly, we identified endothelial cell ($CD31^{+}Sele^{+}Glut1^{+}$) and neutrophil ($Ly6G^{+}Lta4h^{+}Sort1^{+}$) subsets that were closely associated with acute liver dysfunction during sepsis progression. Furthermore, we found that ATF4 inhibition alleviated sepsis-induced acute liver dysfunction, prolonging the survival of septic mice.
Conclusions: These results elucidate the potential mechanisms and subsequent therapeutic targets for the prevention and treatment of sepsis-induced acute liver dysfunction and other liver-related diseases.
Impact and Implications: Sepsis-induced acute liver dysfunction often occurs early in sepsis and can lead to the death of the patient. Nevertheless, the pathogenesis of sepsis-induced acute liver dysfunction is not yet clear. We identified the major cell types associated with acute liver dysfunction and explored their interactions during sepsis. In addition, we also found that ATF-4 inhibition could be invoked as a potential therapeutic for sepsis-induced acute liver dysfunction.
© 2023 The Author(s). Published by Elsevier B.V. on behalf of European Association for the Study of the Liver (EASL). This is an open access article under the CC BY license (http://creativecommons.org/licenses/by/4.0/).

Introduction

Sepsis is characterised by a dysregulated host immune response to trauma or overwhelming infection (*e.g.* severe COVID-19 infection[1]), leading to life-threatening organ dysfunction.[2] Despite numerous advances in medical care, sepsis remains the most common cause of death in intensive care units at present.[3] Acute liver dysfunction often occurs in the early stage of sepsis and has a significant effect on the severity and prognosis of sepsis. To date, specific therapeutic measures in current clinical practice, to resolve sepsis-induced acute liver dysfunction other than liver transplantation, is still lacking.[4] However, the shortage of liver sources as well as lifelong immunosuppression and a high medical cost associated with liver transplantation has resulted in an urgent need for alternative therapeutic interventions that is effective in treating sepsis-induced acute liver dysfunction.

Clarifying the pathogenesis of sepsis-induced acute liver dysfunction will provide opportunities to develop novel therapeutic interventions. Although previous studies have reported that acute liver dysfunction involves a complex interplay between multiple nonparenchymal cell lineages including neutrophils, endothelial cells, and Kupffer cells,[5] their cellular heterogeneity and dynamic regulation leading to acute liver dysfunction remain poorly understood.

Keywords: Sepsis; Single-cell RNA sequencing; Acute liver dysfunction; Activating transcription factor 4.
Received 15 August 2022; received in revised form 14 February 2023; accepted 16 February 2023; available online 1 March 2023
[†] These authors contributed equally to this work.
* Corresponding authors. Addresses: Institute of Health Service and Transfusion Medicine, Academy of Military Medical Sciences, Beijing 100850, China (G. **Chen**; H. Zhou); Translational Medicine Research Center, Fourth Medical Center and Medical Innovation Research Division of the Chinese PLA General Hospital, Beijing 100048, China (Y. Yao).
E-mail addresses: chenlzu2005@163.com (G. Chen), c_ff@sina.com (Y. Yao), zhouhtt1966@163.com (H. Zhou).

ELSEVIER
EASL The Home of Hepatology

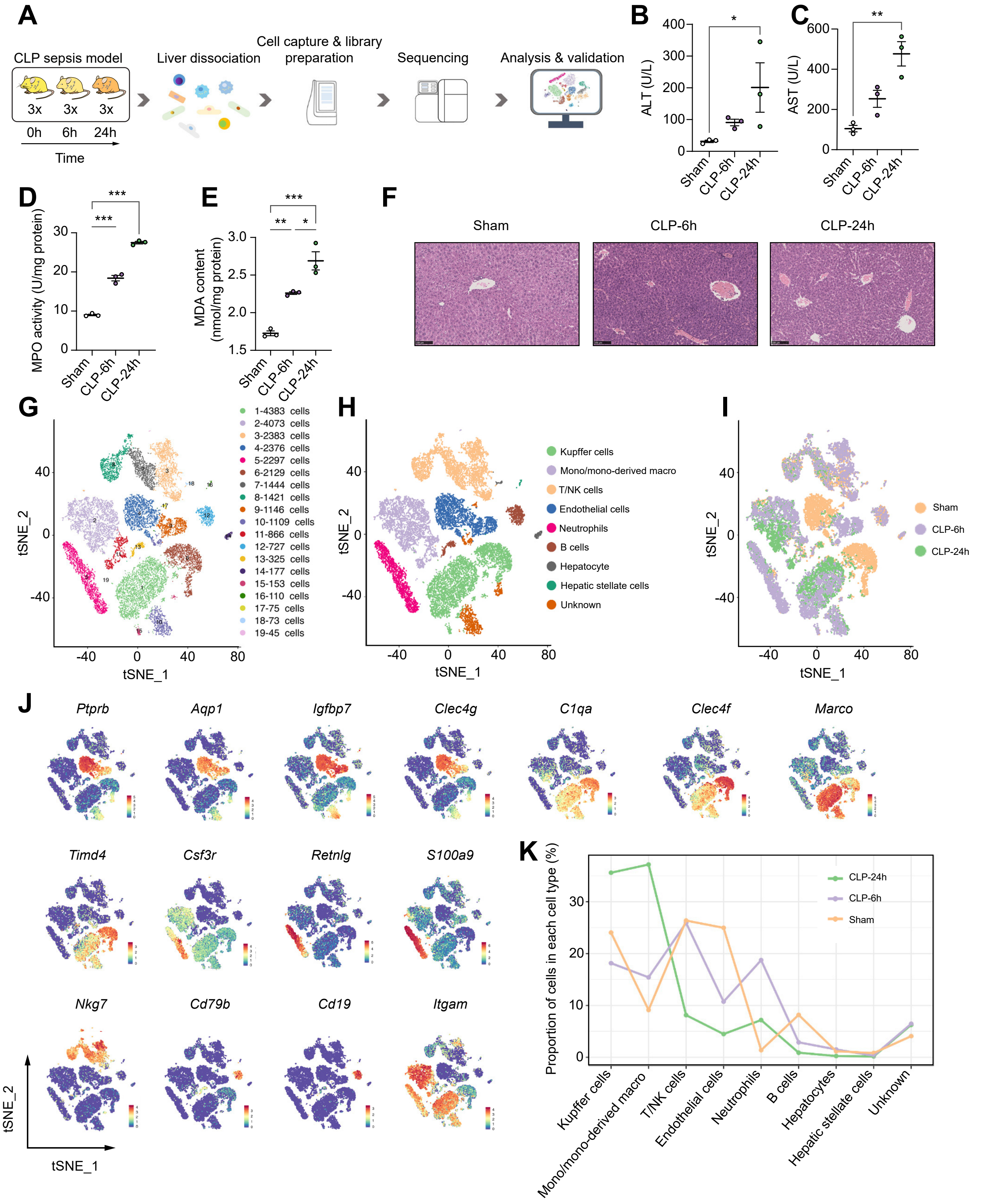

Fig. 1. scRNA-seq identified hepatic cell populations and specific markers in the mice liver. (A) Schematic diagram indicating the procedure for scRNA-seq. For each experimental condition, the hepatic cells were pooled from three mice in each group. (B) The levels of ALT in plasma. (C) The levels of AST in plasma. (D) MPO activity and (E) the MDA content in the liver. (F) Histological injury of liver (scale bar, 100 μm). (G) UMAP plot of hepatic cells, with colours denoting different cell type clusters. (H) UMAP plot of hepatic cells, with colours denoting different clusters. (I) UMAP plot of cell clusters in hepatic cells across the indicated

The rapid development of single-cell RNA sequencing (scRNA-seq) provides high-dimensional information about tissues and an unprecedented understanding of cellular composition, response, and crosstalk in a pathological state.[6,7] The scRNA-seq has been performed to study several liver diseases from mice and humans.[6,8,9] However, most of these studies have focused on chronic liver diseases including liver cirrhosis, non-alcoholic fatty liver disease, and liver cancer,[8,10] and not many have been conducted on acute liver diseases.

Therefore, in this study, we performed a scRNA-seq experiment on livers following sepsis-induced acute liver dysfunction in a clinically relevant polymicrobial sepsis model. Our analyses revealed the major cell types associated with acute liver dysfunction including neutrophils, endothelial cells, and Kupffer cells; identified subclusters of each cell type; and uncovered their dynamic transformations and interactions. Notably, we reported the extensive activating transcription factor 4 (ATF4) activation in major hepatic cells, as well as the therapeutic efficacy of ATF4 inhibition for sepsis and sepsis-induced acute liver dysfunction.

Materials and methods

Animal care, sepsis model, and animal experiment

All experiments and procedures conformed to the National Institutes of Health guidelines, with the approval of the Institutional Animal Care and Use Committee of the Academy of Military Medical Sciences. The male C57BL6/J mice (7–8 weeks) were obtained from Beijing Vital River Laboratories (Beijing, China) and housed according to the following standard laboratory procedures. After a minimum of 3 days of acclimatisation, the caecal ligation and puncture (CLP) sepsis model was induced as described previously.[11] Briefly, mice were anesthetised with pentobarbital sodium (50 mg/kg). After sterilisation, a midline laparotomy was conducted to expose the caecum, which was ligatured and perforated with a sterile 21-gauge needle. Then a small droplet of faeces was extruded from the puncture, and the caecum was returned into the peritoneal cavity. Subsequently, the mice were resuscitated via a s.c. administration of 1 ml of normal saline.

Nine mice were randomly allocated to the control or sepsis groups; the normal group (control check [CK], n = 3) acted as the control group, whereas the sepsis groups were subjected to CLP and sacrificed at 6 (n = 3) or 24 h (n = 3). The livers and blood from mice in the three groups were immediately collected for further processing.

Cell isolation

After being washed twice with cold normal saline, liver tissues from the same group were pooled and minced to <0.5-mm cubic pieces, followed by enzymatic digestion (1 h at 37 °C) with manual shaking every 5 min. After filtering, dead cell removal, and lysis of red blood cells, the hepatic cells were counted using BD Rhapsody™ Scanner (BD Biosciences, San Jose, CA, USA).

Single-cell RNA sequencing

Single-cell libraries were prepared using the BD Rhapsody Single-Cell Analysis System (BD Biosciences) following the manufacturer's guidelines. Libraries were sequenced using multiple runs on an Illumina NovaSeq 6000 platform (Illumina, San Diego, CA, USA) in a 2 × 150 bp paired-end mode.

Sequencing data analysis

The obtained RNA-sequencing data were processed into the expression matrix Fastq via the BD Rhapsody Analysis Pipeline (v1.9). After quality control, normalisation, and batch correction using the R package 'Seurat' and fastMNN, BD DataView software (BD Biosciences) and the R package 'Seurat' (v3.1.1) were used to analyse the expression matrix.

To reduce the dimensionality of all data, principal component analysis was conducted in Seurat. For t-distributed stochastic neighbour embedding (tSNE) projection and clustering analysis, the cells were clustered based on a graph-based clustering approach and visualised in two dimensions using tSNE. The FindAllMarkers function in Seurat was used to identify the marker genes of each cluster, and the FindMarkers function was used to analyse the differentially expressed genes. A p value <0.05 and $|\log_2$ fold change$|$ >0.58 were set as thresholds for significantly differential expression.

The Kyoto Encyclopedia of Genes and Genomes (KEGG) analysis was performed using the R package 'enrichplot'. Cell–cell interaction analysis was performed using the CellChat R package (v1.1.3).

Single-cell regulatory network inference and clustering (SCENIC) analysis was conducted using the motif database for RcisTarget and GRNboost (SCENIC v1.1.2.2, which corresponds to AUCell v1.4.1 and RcisTarget v1.2.1). The 'RcisTarget' package was used to identify transcription factor (TF) binding motifs and potential target genes (regulons). The activity of the regulon group in each cell was classified using the 'AUCell' package. The regulon specificity scores for each cell type were calculated using the 'scFunctions' package.

Drug administration

To evaluate the therapeutic effect of integrated stress response inhibitor (ISRIB), a total of 28 mice were randomly divided into the control or experimental groups; the normal group (CK, n = 3) and the ISRIB-treated group (5 mg/kg, n = 3) acted as controls, whereas the experimental groups were i.p. treated with ISRIB (5 mg/kg, n = 11) or an equal volume of vehicle (n = 11) 2 h before CLP. Mice were sacrificed 24 h after CLP, and the livers and plasma were collected and stored until assayed.

Blood biochemistry and bloodocyte analysis

Blood biochemistry was analysed using a biochemical auto analyser (Pointcare V2, MNCHIP, Tianjin, China). Complete blood counts were analysed using a BC-5000 Vet auto haematology analyser (Mindray, Shenzhen, China).

conditions. (J) The markers indicating group identities. (K) The proportion of hepatic cell populations in each sample. *p <0.05; **p <0.01; ***p <0.001. Statistical differences between groups were assessed using one-way ANOVA for (B)–(E). ALT, alanine aminotransferase; AST, aspartate aminotransferase; CLP, caecal ligation and puncture; CLP-24 h, 24 h after CLP; CLP-6h, 6 h after CLP; MDA, malondialdehyde; MPO, myeloperoxidase; scRNA-seq, single-cell RNA sequencing; T/NK, T/natural killer; tSNE, t-distributed stochastic neighbour embedding; UMAP, uniform manifold approximation and projection.

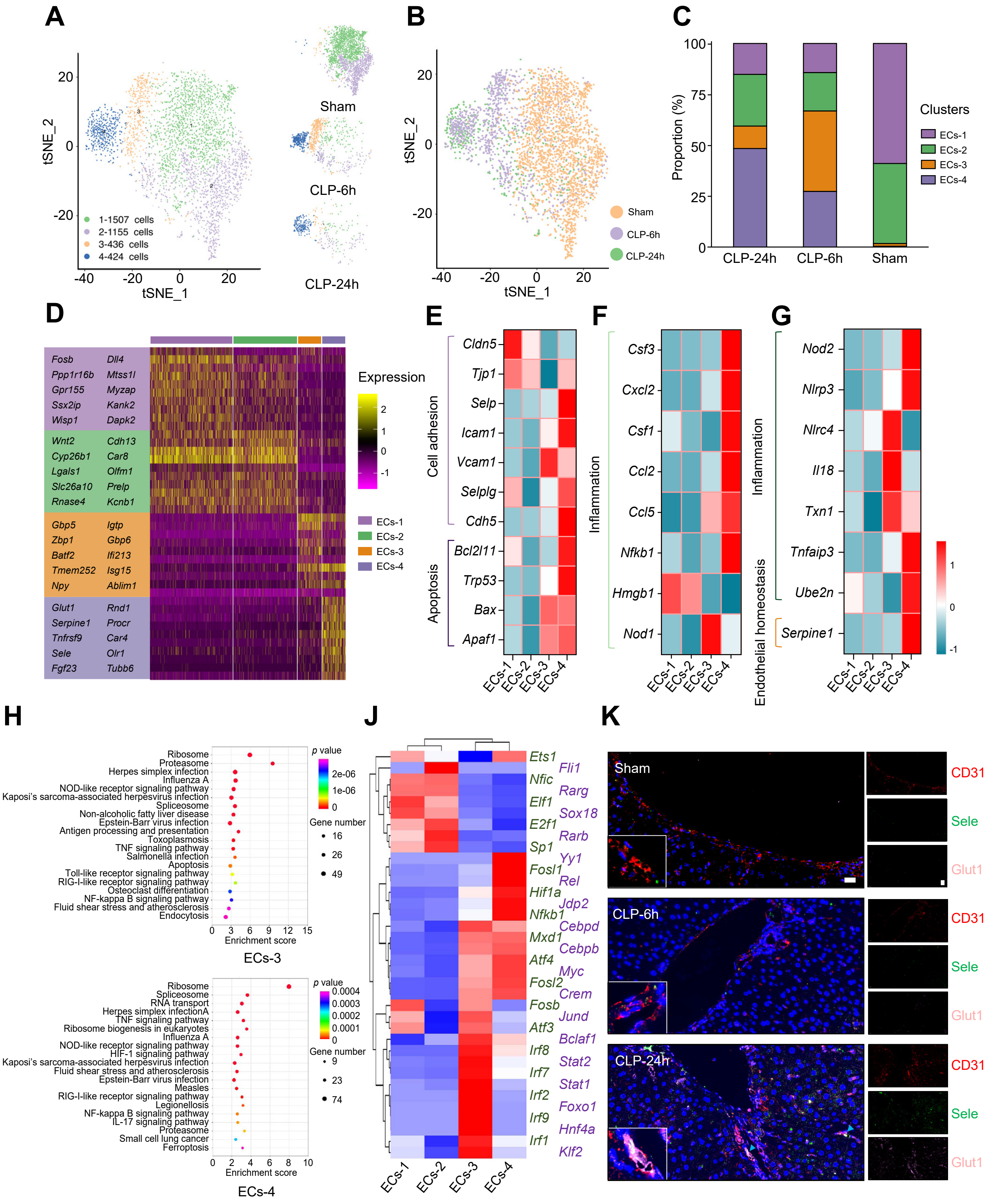

Fig. 2. Hepatic endothelial cell dynamics and functionality in healthy and septic livers. (A) Subclustering of endothelial cells in healthy and septic livers. (B) UMAP plot of endothelial cells across the indicated conditions. (C) The proportion of endothelial cell subclusters in each sample. (D) Heatmap plots demonstrating expression of specified genes among endothelial cell subclusters. (E–G) Heatmap showing gene expression by the indicated endothelial cell subclusters. (H) The enriched pathway associated with ECs-3 and ECs-4. Colours indicate significance of enrichment, and circle sizes indicate number of genes falling into respective

Lipid peroxidation, neutrophil infiltration, and IL-6 content in livers

The livers were homogenised in ice-cold saline (Shijiazhuang Siyao Ltd., Hebei, China) and centrifuged (1000×g, 6 min, 4 °C). The obtained homogenates were then assayed for malondialdehyde (MDA) content, myeloperoxidase (MPO) activity, and IL-6 level, according to the manufacturer's instructions, as described previously.[12]

Histological analysis and immunofluorescence

The paraformaldehyde-fixed livers were dehydrated, embedded in paraffin, and cut into 5- to 7-μm-thick sections before being subjected to H&E staining as described previously.[12] The severity level of liver damage was assessed for inflammatory infiltration, cell swelling, and tissue architecture disruption in a blinded fashion and scored on a 4-point scale (0, none; 1, slight; 2, moderate; 3, severe).

For immunofluorescence assays, the liver sections were incubated with anti-CD31 (1:3,000, Proteintech, IL, USA), anti-Glut1 (1:100, Proteintech), anti-Sele (1:2,000, Proteintech), anti-Ly6G (1:3,000, Servicebio, Wuhan, China), anti-Lta4h (1:1,000, Proteintech), anti-Sort1 (1:400, Proteintech), anti-ATF4 (1:1,000, ABclonal, Wuhan, China), anti-Fosl1 (1:1,000, ABclonal), anti–NF–κB1(1:1,000, ABclonal), and anti-F4/80 (1:500, Servicebio) primary antibodies, followed by washing and incubation with the fluorophore-labelled secondary antibody, and visualisation using a confocal microscope (Nikon, Tokyo, Japan).

Survival experiments

A total of 100 mice were randomised into the normal control or sepsis model groups. The sepsis model groups were i.p. treated with ISRIB (8 mg/kg, n = 45) or an equivalent volume of vehicle (n = 45) 2 h before CLP. The normal mice acted as the control group.

Statistical analysis

Data are shown as mean ± SEM. Statistical differences between groups were determined using one-way ANOVA or unpaired Student's *t* test. Survival data were analysed using the log-rank test. $^{*}p$ <0.05, $^{**}p$ <0.01, and $^{***}p$ <0.001 were considered the thresholds for statistical significance of differences.

Results

scRNA-seq identified multiple cell populations in the liver from control and sepsis mice

To elucidate diverse hepatic nonparenchymal cell types and comprehensively characterise their dynamic changes during the pathological progression of sepsis-induced acute liver dysfunction, we performed scRNA-seq analysis of the livers at different time points (6 and 24 h) after sepsis using the BD Rhapsody platform (Fig. 1A). These two time points reflected the major stages in the development of sepsis-induced acute liver dysfunction, corresponding to a progressive increase in liver injury. As shown in Fig. 1B and C, the plasma levels of alanine aminotransferase (ALT) and aspartate aminotransferase (AST), which are used as biomarkers for acute liver dysfunction,[13] gradually became elevated over time. Similarly, MDA content, MPO activity, and histological injury in the liver also increased gradually with time, indicating that sepsis could induce lipid peroxidation and neutrophil infiltration during its pathological progression (Fig. 1D–F). In addition, the sepsis model mice also exhibited acute kidney injury and had a low level of glucose and total protein in blood (Fig. S1A–D).

A total of 27,972 liver nonparenchymal cells and hepatocytes from control and sepsis mice were further analysed after passing quality control metrics and having corrected for batch effect. Eight major cellular clusters consisting of 19 clusters (marker genes shown in Fig. S1F) were identified (Fig. 1G–J), including endothelial cells (*Ptprb*, *Aqp1*, *Igfbp7*, *Clec4g*, *Ehd3*, *Ushbp1*, *Oit3*, *Il1a*, *F8*, *Bmp2*, *C1qtnf1*, *Mmrn2*, *Pcdh12*, and *Dpp4*), Kupffer cells (*C1qa*, *C1qb*, *C1qc*, *Clec4f*, *Csf1r*, *Adgre1*, *Clec4f*, *Irf7*, *Spic*, *Timd4*, and *Marco*), neutrophils (*Csf3r*, *Retnlg*, *S100a8*, *S100a9*, *Slpi*, *Mmp9*, *Mmp8*, and *Adam8*), T/natural killer cells (*Nkg7*), B cells (*Igkc*, *Cd22*, *Cd79b*, *Cd19*, *Cd79a*, *Ebf1*, and *Pax5*), monocytes/monocyte-derived macrophages (*Itgam* and *Ccr2*), hepatocytes (*Alb*, *Cyp2e1*, *Apob*, *Asgr1*, *Pck1*, *Hp*, *Ass1*, and *Apoe*), and hepatic stellate cells (*DCN*, *Hgf*, *Col14a1*, *Col1a1*, *Col1a2*, *Col3a1*, *Colec11*, *Cxcl12*, and *Cygb*).

In addition, the relative composition of different cell types across different stages of disease and healthy controls was investigated to uncover substantial changes during disease progression. A rapid increase of the neutrophil population was observed 6 h after CLP when compared with that of controls. Subsequently, the proportion of neutrophils declined at 24 h after CLP compared with that at 6 h after CLP (Fig. 1K). The relative proportion of the endothelial and B cells decreased with pathological progression, whereas that of monocytes/monocyte-derived macrophages increased with pathological progression. In addition, there was a slight decrease in the proportion of Kupffer cell population at 6 h after CLP compared with that of controls, but this proportion robustly increased at 24 h after CLP, which indicated a rapid supplement of Kupffer cells from blood circulation. These results suggested that different hepatic non-parenchymal cell types, especially immune populations changed dynamically at different time points and reacted collectively to acute liver dysfunction via distinct functions.

Pecam1(CD31), Adgre1(F4/80), and Ly6g have been reported as cell makers of endothelial cells, Kupffer cells, and neutrophils, respectively.[14–17] The tSNE plots for *Pecam1*, *Ly6g*, and *Adgre1* expressions in all cell clusters are shown in Fig. S1E. Our results show that regions with high *Ly6g*, *Pecam1*, and *Adgre1* expression are strongly correlated with the neutrophils, endothelial cells, and Kupffer cells, respectively.

scRNA-seq revealed disease-specific endothelial cell subpopulations

Previous studies have highlighted liver endothelial cells that orchestrate the progression of liver injury.[8,18] Thus, further

categories. (J) Subpopulation-specific regulons of each subpopulation revealed by SCENIC analysis. (K) Immunofluorescence staining results showing the spatial distribution of the ECs-4 subpopulations in healthy and septic mouse livers. Scale bars, 20 μm. CLP, caecal ligation and puncture; CLP-24 h, 24 h after CLP; CLP-6h, 6 h after CLP; ECs-1 to ECs-4, endothelial cell subclusters 1 to 4; NOD, nucleotide oligomerisation domain; SCENIC, single-cell regulatory network inference and clustering; TNF, tumour necrosis factor; tSNE, t-distributed stochastic neighbour embedding; UMAP, uniform manifold approximation and projection.

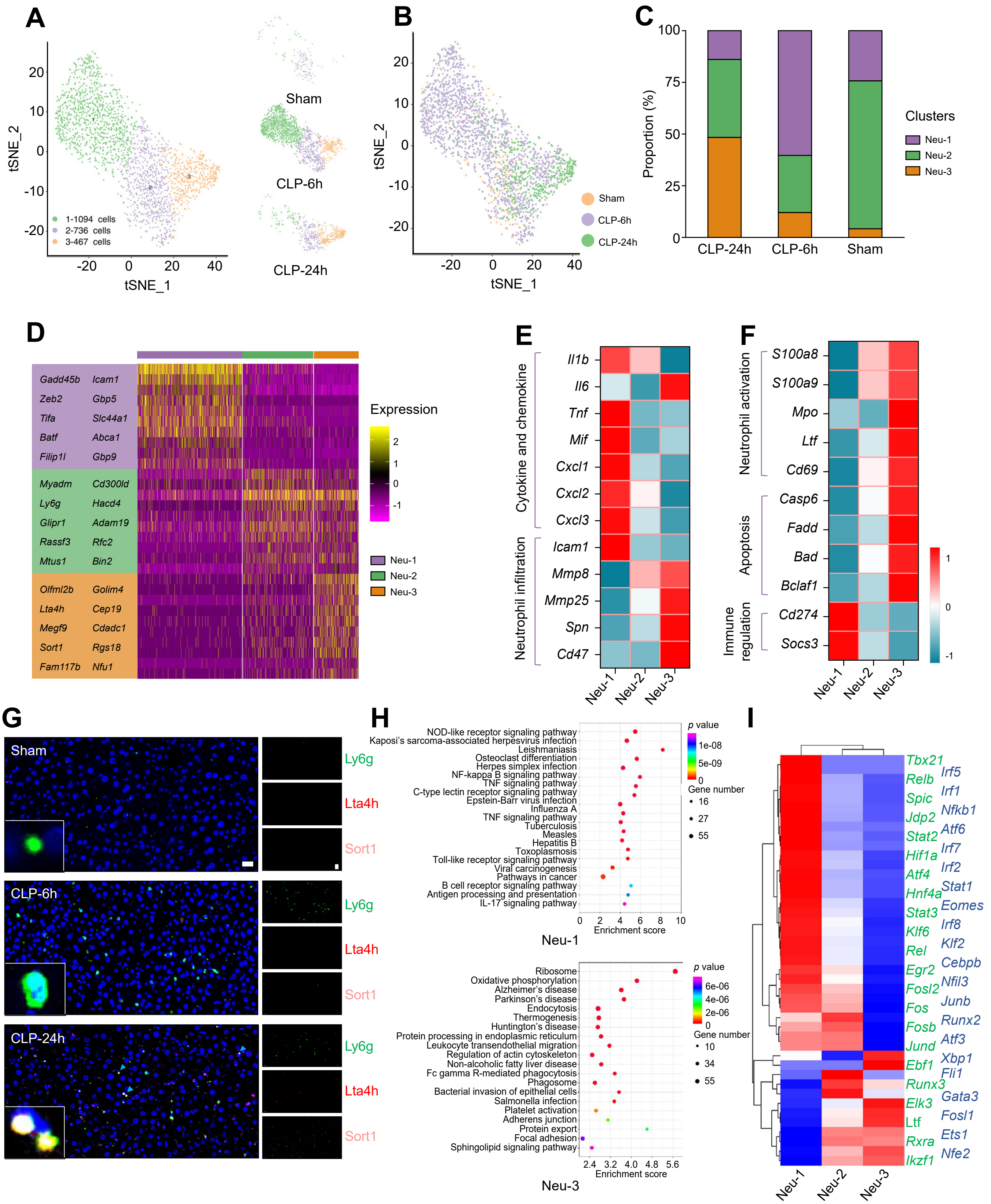

Fig. 3. Hepatic neutrophil dynamics and functionality in healthy and septic livers. (A) Subclustering of neutrophils in healthy and septic livers. (B) UMAP plot of neutrophils across the indicated conditions. (C) The proportion of neutrophil subclusters in each sample. (D) Heatmap plots demonstrating expression of specified genes among neutrophil subclusters. (E and F) Heatmap showing gene expression by the indicated neutrophil subclusters. (G) Immunofluorescence staining results showing the spatial distribution of the Neu-3 subpopulations in healthy and septic mouse livers. Scale bars, 20 μm. (H) The enriched pathway

analysis on our scRNA-seq data was performed to investigate the transcriptional diversity of endothelial cells in liver.

The liver endothelial cells were grouped into four subclusters (Fig. 2A and B); ECs-1 and ECs-2 accounted for up to 98.44% of liver endothelial cells under healthy conditions (Fig. 2C), whereas endothelial cells in ECs-3 and ECs-4 were nearly exclusively present in the livers of sepsis mice, and the proportion of endothelial cells in ECs-4 increased with the pathological progression of sepsis-induced acute liver dysfunction (Fig. 2C), indicating that the ECs-4 subcluster represented a unique population of endothelial cells associated with the pathogenesis of sepsis-induced acute liver dysfunction. Based on a subsequent gene enrichment analysis, ECs-3 was found differentially expressing *Zbp1*, *Batf2*, and *Isg15*. Zbp1 can act as a DNA sensor and mediate the recruitment of RIP1 and RIP3 to activate the NF-κB and NLRP3 inflammasome pathways.[19,20]

In contrast, ECs-4 showed the highest expression of *Glut1*, *Serpine1*, *Sele*, *Fgf23*, *Car4*, *Rnd1*, and *Tnfrsf9* (Fig. 2D and Fig. S2C). Among these highly expressed genes of ECs-4, the expression of *Serpine1*, *Sele*, and *Fgf23* is associated with endothelial dysfunction.[18,21,22] *Tnfrsf9* expression could exacerbate inflammation through promoting cell adhesion and leucocyte chemoattraction.[22] Similarly, the increase of *Rnd1* expression could induce actin cytoskeletal rearrangement of endothelium, thereby aggravating the inflammatory response.[23] This evidence further indicates that the dysfunction of ECs-4 leads to the acceleration of sepsis-induced acute liver dysfunction.

In addition, ECs-3 showed an upregulation of inflammation-associated genes (*Il18*, *Nlrc4*, *Txn1*, and *Nod1*) (Fig. 2F and G). As for cell adhesion molecules, ECs-3 exhibited a relatively high expression of *Vcam-1* and a low expression of *Tjp1* and *Cldn5* (Fig. 2E), indicating poor cellular adhesion of endothelial cells in ECs-3. The ECs-4 subcluster also showed an upregulation of genes related to inflammation (*Nlrp3*, *Tnfaip3*, *Ube2n*, *Cxcl2*, *Ccl2*, *Ccl5*, *Csf3*, *Nfkb1*, and *Nod2*) (Fig. 2F and G), cell adhesion (*Cdh5*, *Selplg*, *Icam1*, and *Selp*) (Fig. 2E), and apoptosis (*Apaf1*, *Bax*, *Trp53*, and *Bcl2l11*) (Fig 2E). Interestingly, ECs-4 exhibited a low level of *Hmgb1* (Fig. 2F). Hmgb1 is considered an important mediator in the pathogenesis of sepsis-induced acute liver dysfunction.[24,25] KEGG pathway analyses revealed that the genes that were upregulated in ECs-3 belonged to the Toll-like receptor, antigen processing and presentation, and nucleotide oligomerisation domain (NOD)-like receptor signalling pathways (Fig. 2H), whereas the genes that were upregulated in ECs-4 belonged to the tumour necrosis factor (TNF)/NF-κB, IL-17, and NOD-like receptor signalling pathways (Fig. 2H and Fig. S2A and B).

Furthermore, SCENIC analysis was used to assess the expression status of TFs in different endothelial cell subpopulations. As shown in Fig. 2J, the genes regulated by the Yy1, Fosl1, Hif1α, and Atf4 TFs were upregulated in ECs-4. Notably, Atf4 is involved in the complex process of cellular stress response[26,27] and can be used as a pharmacological target in several diseases including diabetes mellitus,[28] atrial fibrillation,[29] and age-related memory decline.[30] High endothelial Hif-1α expression promoted CXCL1 expression and monocyte adhesion to endothelial cells,[31] and Yy1 expression was correlated with the inflammatory NK-κB activity and neutrophil infiltration and subsequently resulted in enhanced inflammatory effects.[32,33] Fosl1 is part of the activator protein complex, which makes up the TF activator protein 1 (AP-1). AP-1 has been suggested to be responsible for activating inflammation.[34]

To confirm the emergence of ECs-4 during the pathogenesis of sepsis-induced acute liver dysfunction, the immunofluorescence staining was performed using CD31, Sele, and Glut1 antibodies to detect this endothelial cell subpopulation (Fig. 2K). Consistent with single-cell analysis, the proportion of $CD31^{+}Sele^{+}Glut1^{+}$ endothelial cells increased gradually, concomitant with the development of acute liver dysfunction. In addition, total endothelial cells were detected using CD31 immunofluorescence staining. Our results indicate that the number of total endothelial cells decrease with pathological progression, being consistent with the scRNA-seq result (Fig. S2D).

Altogether, the analysis of endothelial cells indicated that hepatic endothelial cells exhibited heterogeneity, and differences among subtypes with distinct gene expression patterns represented their unique functionality during sepsis. Here, the unique subpopulation of endothelial cells with endothelial dysfunction-related genetic characteristics was also identified and is speculated to be closely related to the pathology of sepsis-induced acute liver dysfunction.

scRNA-seq revealed heterogeneity of neutrophils in liver during sepsis

Neutrophils, which are well characterised to be recruited rapidly at sites of infection, function as first-line responders specialised in elimination of invading pathogens. However, simultaneously exaggerated activation and uncontrolled tissue infiltration of neutrophils cause excess oxidative stress, inflammation, and subsequent tissue injury,[35] thus making them a potential therapeutic target to treat sepsis in our study and others.[13,36] However, neutrophil populations in sepsis are not homogenous; therefore, exploring neutrophil heterogeneity will help regulate neutrophils accurately to obtain a balance between protective immunity and tissue injury.

The hepatic neutrophils were further grouped into three subclusters: Neu-1 (mainly expressing *Gadd45b*, *Icam1*, *Gbp5*, and *Tifa*), Neu-2 (mainly expressing *Myadm*, *Cd300ld*, and *Ly6g*), and Neu-3 (mainly expressing *Lta4h*, *Sort1*, and *Rgs18*) (Fig. 3A, B, and D, and Fig. S3A). Neu-2 was the major subcluster of neutrophils and accounted for up to 71.54% of liver neutrophils in healthy mice (Fig. 3C). Neu-1 was the major subcluster (60.61%) of neutrophils at the early stage of CLP (6 h), whereas the neutrophils in Neu-3 constituted the major subcluster (48.16%) of neutrophils at 24 h after CLP. Impressively, the proportion of Neu-3 in the liver exhibited a continuous increase in a time-dependent manner during sepsis, which were verified via immunofluorescence staining of Ly6G, Lta4h, and Sort1 in healthy and sepsis mice (Fig. 3G). In addition, our results indicate that the changes in the number of total neutrophils in different groups were consistent with the scRNA-seq result (Fig. S3B).

associated with Neu-1 and Neu-3. Colours indicate significance of enrichment and circle sizes indicate number of genes falling into respective categories. (I) Subpopulation-specific regulons of each subpopulation revealed by SCENIC analysis. CLP, caecal ligation and puncture; CLP-24 h, 24 h after CLP; CLP-6h, 6 h after CLP; Neu-1 to Neu-3, neutrophil subclusters 1 to 3; NOD, nucleotide oligomerisation domain; SCENIC, single-cell regulatory network inference and clustering; TNF, tumour necrosis factor; tSNE, t-distributed stochastic neighbour embedding; UMAP, uniform manifold approximation and projection.

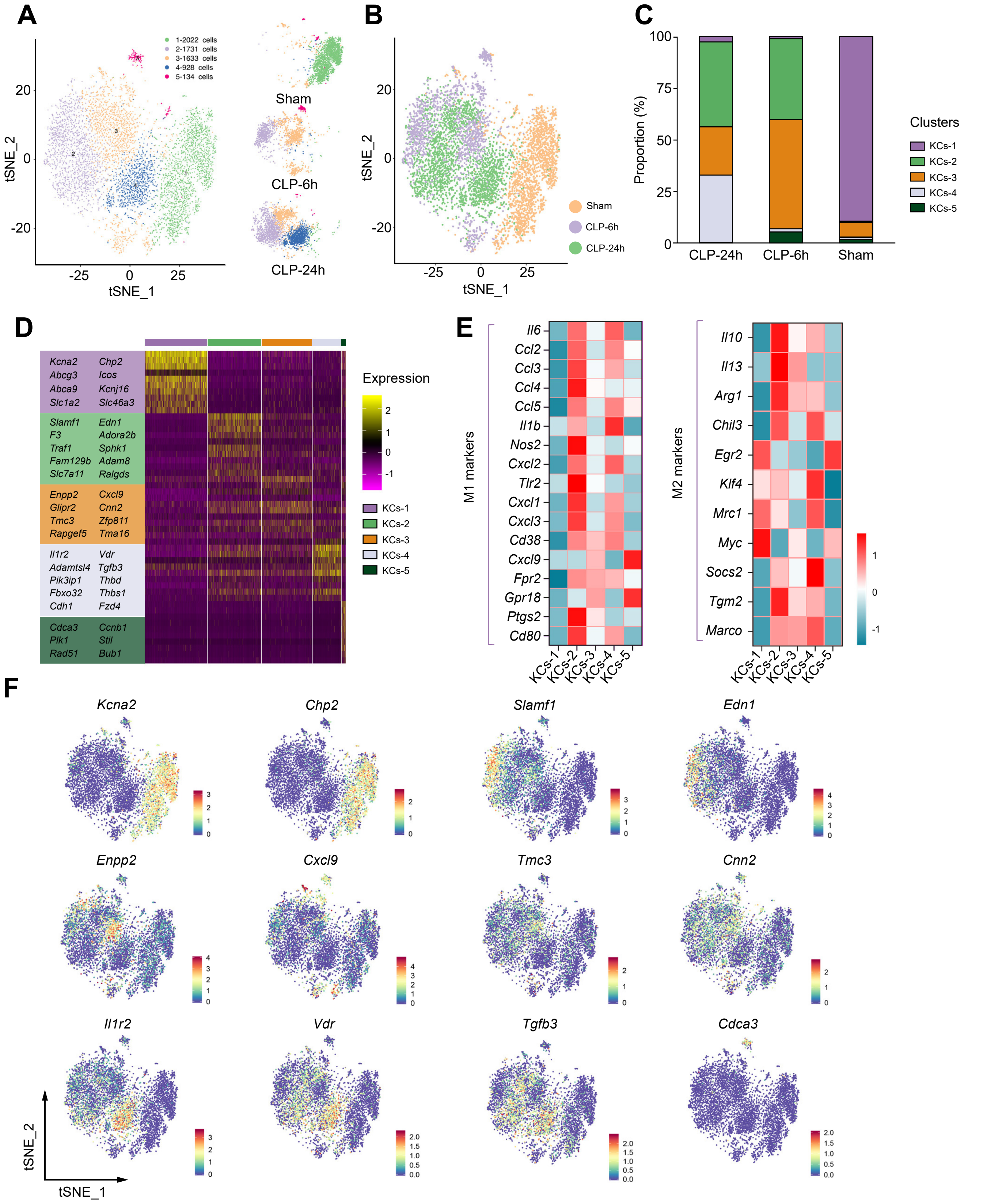

Fig. 4. Hepatic Kupffer cell dynamics and functionality in healthy and septic livers. (A) Subclustering of Kupffer cells in healthy and septic livers. (B) UMAP plot of Kupffer cells across the indicated conditions. (C) The proportion of Kupffer cell subclusters in each sample. (D) Heatmap plots demonstrating expression of specified genes among Kupffer cell subclusters. (E) Heatmap showing gene expression by the indicated Kupffer cell subclusters. (F) UMAP plot showing subcluster-specific marker genes of Kupffer cells. CLP, caecal ligation and puncture; CLP-24 h, 24 h after CLP; CLP-6h, 6 h after CLP; KCs-1 to KCs-4, Kupffer cell subclusters 1 to 4; tSNE, t-distributed stochastic neighbour embedding; UMAP, uniform manifold approximation and projection.

Further analysis revealed that neutrophil subclusters exhibited different transcriptional characteristics (Fig. 3E and F). Both Neu-1 and Neu-3 were inflammatory neutrophils that bore a pro-inflammatory gene signature. Neu-1 showed a high expression of pro-inflammatory cytokines (*Il1b*, *Tnf*, and *Mif*), neutrophil-attracting chemokines (*Cxcl1*, *Cxcl2*, and *Cxcl3*), and adhesion molecule (*Icam1*) (Fig. 3E). Notably, Neu-1 also exhibited a high expression of immune regulatory genes (*Cd274* and *Socs3*) (Fig. 3F). Neu-3 showed a hyperactivated phenotype with a high expression of calgranulins (*S100a9* and *S100a8*), neutrophil activation markers (*Mpo*, *Ltf*, and *Cd69*), neutrophil infiltration-related genes (*Cd47*, *Spn/Cd43*, *Mmp8*, and *Mmp25*), and pro-inflammatory cytokines (*Il-6*) (Fig. 3E and F). In addition, Neu-3 showed apoptotic properties via the upregulation of the pro-apoptosis-related genes (*Fadd*, *Casp6*, *Bad*, and *Bclaf1*) (Fig. 3F). Collectively, it is suspected that Neu-1 responded to the microbial invasion in the liver, and became activated to eliminate microbial organisms, while concomitantly releasing inflammatory cytokines and chemokines to recruit more immune cells, including Neu-3, to the liver. Consequently, a significant number

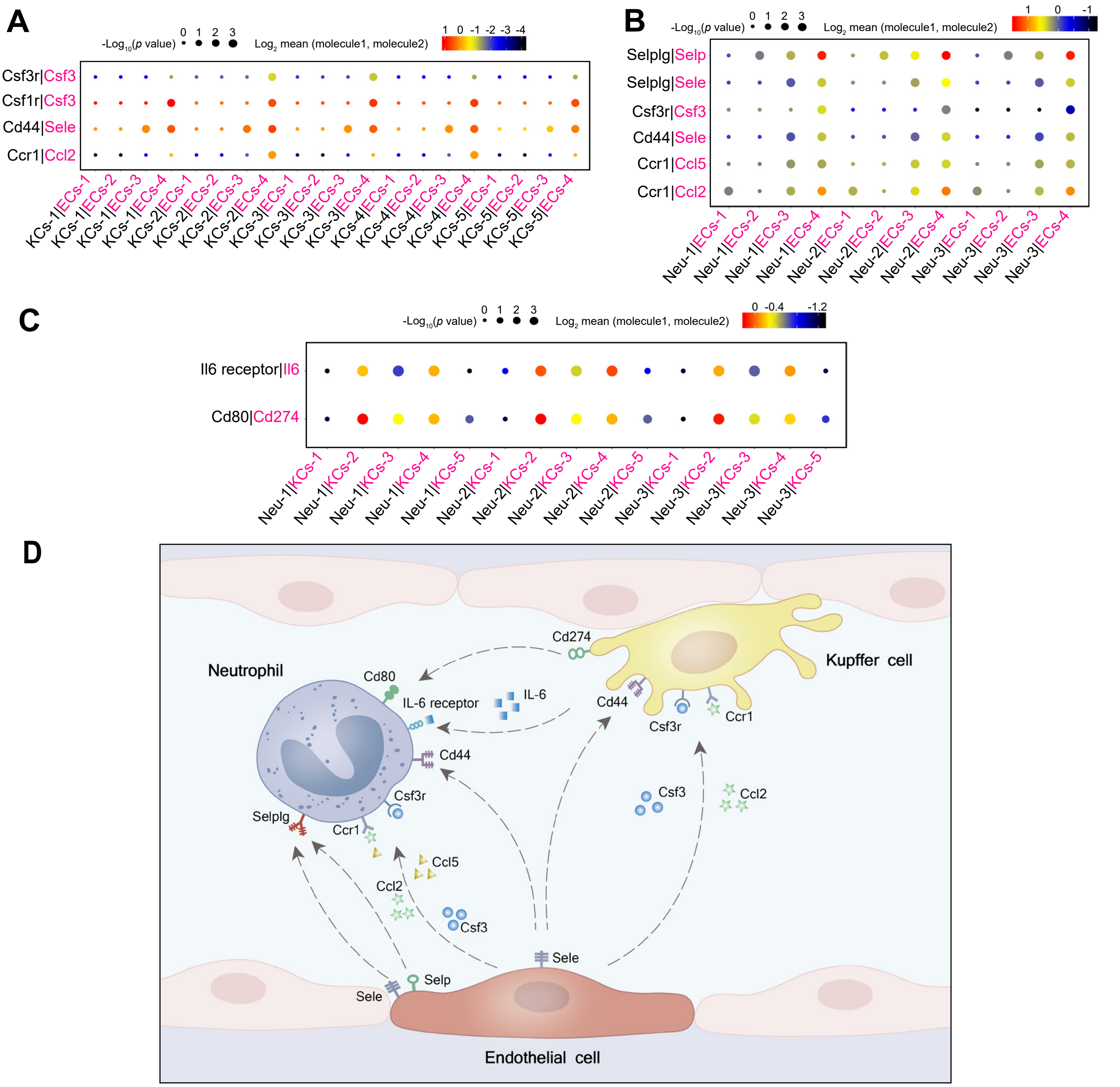

Fig. 5. Molecular interactions of neutrophils, endothelial cells, and Kupffer cells. (A) Interactions between four endothelial cell subclusters and five Kupffer cell subclusters. (B) Interactions between three neutrophil subclusters and four endothelial cell subclusters. (C) Interactions between three neutrophil subclusters and four Kupffer cell subclusters. Dot sizes and colours represent logarithmic-transformed *p* values and mean expression of interacting molecules in corresponding cells. (D) Predicted main regulatory network among endothelial cells, neutrophils, and Kupffer cells during sepsis. ECs-1 to ECs-4, endothelial cell subclusters 1 to 4; KCs-1 to KCs-5, Kupffer cell subclusters 1 to 5; Neu-1 to Neu-3, neutrophil subclusters 1 to 3.

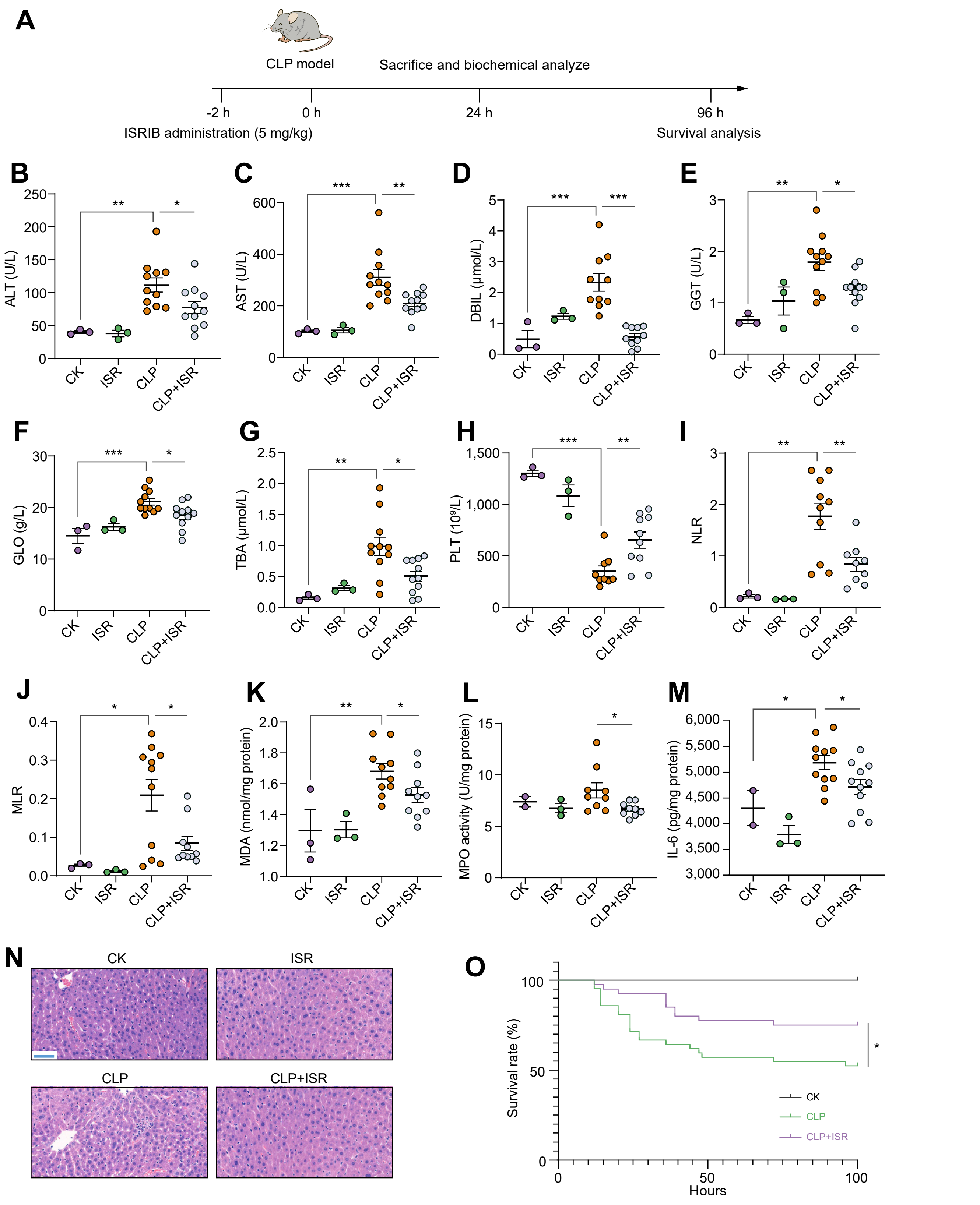

Fig. 6. Therapeutic effects of ATF4 inhibition on sepsis-induced acute liver dysfunction and survival in CLP-induced sepsis model. (A) Experimental timeline of ATF4 inhibition in CLP-induced sepsis model (*in vivo*). The levels of (B) ALT, (C) AST, (D) DBIL, (E) GGT, (F) GLO, and (G) TBA in plasma 24 h after CLP. The (H) PLT in blood, (I) NLR, and (J) MLR. The (K) MDA content, (L) MPO activity, and (M) IL-6 in liver 24 h after CLP. (N) Representative images show the effect of ISRIB administration on histological injury (scale bar, 50 μm). (O) Survival rates of sepsis mice after 96 h following ISRIB administration. $^{*}p$ <0.05; $^{**}p$ <0.01; $^{***}p$ <0.001.

of activated Neu-3 with robust pathogen removal capability could have infiltrated the liver and eliminated the invading microbial organisms via phagocytosis and the release of cytotoxic antimicrobial molecules (reactive oxygen species and lactoferrin), ultimately leading to liver injury.

KEGG pathway analyses revealed that the genes that were upregulated in Neu-1 belonged to the NOD-like receptor, TNF/NF-κB, C-type lectin receptor, and Toll-like receptor signalling pathways (Fig. 3H). Meanwhile, the genes that were upregulated in Neu-3 belonged to the leucocyte transendothelial migration, endocytosis, and phagosome (Fig. 3H).

SCENIC analysis was performed to assess which TFs were responsible for the differences in gene expression between different cell clusters. *Hif1a*, *Atf4*, *Atf6*, *Irf1*, *Nfkb1*, and *Irf5* were identified as candidate TFs underlying the gene signature of Neu-1 (Fig. 3I), whereas *Xbp1*, *Fosl1*, and *Ltf* were the candidate TFs underlying the differential gene expression in Neu-3.

scRNA-seq revealed heterogeneity of Kupffer cells in liver during sepsis

Kupffer cells represent the major fraction of liver macrophages[37] and play a pivotal role in maintaining homoeostasis of the liver as well as in contributing to the progression of acute liver dysfunction.[38] In this study, Kupffer cells were grouped into five clusters (Fig. 4A, B, D, and F), which were annotated as KCs-1 (enriched in the expression of *Kcna2*, *Abcg3*, and *Slc1a2*), KCs-2 (enriched in the expression of *Slamf1*, *Edn1*, *Adora2b*, and *Adam8*), KCs-3 (enriched in the expression of *Enpp2*, *Cxcl9*, *Tmc3*, and *Cnn2*), KCs-4 (enriched in the expression of *Il1r2*, *Vdr*, *Tgfb3*, and *Cdh1*), and KCs-5 (enriched in the expression of *Cdca3*, *Stil*, and *Rad51*). KCs-1 constituted up to 89.48% of Kupffer cells under healthy conditions, whereas KCs-3 constituted only 7.29% of the total Kupffer cells (Fig. 4C). During the early stage of sepsis (6 h), the proportions of KCs-2 and KCs-3 were increased and accounted for 92.21% of all Kupffer cells. As liver injury progressed in severity, the proportion of KCs-2 increased slightly (39.20 to 40.93%), and the proportion of KCs-3 decreased considerably (53.01 to 23.61%) at 24 h after CLP. Importantly, KCs-4 increased from less than 2% of total Kupffer cells in the sham and CLP-6h groups to 32.57% in the CLP-24 h group. Based on the dynamic changes in the proportion of Kupffer cell subclusters, it is suspected that KCs-2, KCs-3, and KCs-4 play important roles in the development of sepsis-induced acute liver dysfunction. In addition, total Kupffer cells were detected using F4/80 immunofluorescence staining. Our results indicate that the changes in the number of F4/80 positive cells in different groups were consistent with the scRNA-seq result (Fig. S4).

Further analysis revealed that KCs-1, KCs-2, KCs-3, and KCs-4 exhibited different expression profiles of inflammation-related genes (Fig. 4E). KCs-2 showed not only a high expression of pro-inflammatory genes/M1 markers (*e.g. Il-6*, *Ccl2*, *Ccl3*, *Ccl4*, *Nos2*, *Cxcl1*, *Cxcl2*, *Cxcl3*, *Tlr2*, *Ptgs2*, *Fpr2*, *Cd80*, and *Cd38*) but also a high expression of several feedback inhibitors of activation (*Il10*, *Il13*, *Arg1*, and *Tgm2*). KCs-3 also exhibited a high expression of pro-inflammatory genes, specifically *Cxcl9*, *Fpr2*, and *Gpr18*. Similarly, KCs-4 showed a relatively high expression of pro-inflammatory genes (*Il-6*, *Ccl2*, *Ccl3*, *Ccl5*, *Il-1b*, *Cd38*, *Cxcl2*, and *Fpr2*), as well as genes associated with M2-like Kupffer cells (*Il10*, *Mrc1*, *Socs2*,[39] *Arg1*, *Klf4*,[40] and *Chil3*[41]). In contrast, KCs-1 exhibited a very low expression of pro-inflammatory genes and a high expression level in genes associated with M2-like Kupffer cells (*Myc* and *Egr2*). These results indicated that KCs-2, KCs-3, and KCs-4 were activated and played a vital role in releasing cytokines and chemokines, which most likely activated and recruited circulating macrophages and neutrophils to liver tissue, subsequently leading to liver injury.

Cell-to-cell communication in CLP-induced liver dysfunction: receptor–ligand analysis

Elucidating the explicit interaction among liver cells during sepsis will shed light on the pathogenesis of sepsis-induced acute liver dysfunction. The ligand–receptor pairs among the major cell types are shown in Fig. S5. Notably, the Kupffer cells showed the most interactions with other cell types during sepsis, in particular with endothelial cells and neutrophils, at 24 h after sepsis. To further explore the detailed cellular communication during CLP-induced liver injury, the intercellular interactions within heterogeneous populations of neutrophils, Kupffer cells, and endothelial cells were analysed. As shown in Fig. 5A, ECs-4, the major subcluster of endothelial cells at 24 h after CLP, showed enhanced interactions with Kupffer cells via the Csf3r/Csf3, Csf1r/Csf3, CD44/Sele, and Ccr1/Ccl2 axes (Fig. 5A). Furthermore, endothelial cell–neutrophil interactions are crucial for neutrophil infiltration to the liver.[13] Notably, ECs-4, the major subcluster of endothelial cells at 24 h after CLP, expressed relatively high levels of chemokines (*Ccl2* and *Ccl5*), cytokines (*Csf3*/*G-CSF*), and adhesion molecules (*Sele* and *Selp*), whereas the corresponding receptors were widely expressed in Neu-3, suggesting that functional interactions between Neu-3 and ECs-4 may play significant roles in enhancing neutrophil infiltration into liver tissues (Fig. 5B). In addition, the enhanced interactions between Kupffer cells (KCs-2, KCs-3, and KCs-4) and neutrophils, such as Il6 receptor/Il6 and Cd80/Cd274, were the most noticeable (Fig. 5C).

Our results found that the activated endothelial cells secrete colony-stimulating factors (Csf3) and chemokines (Ccl2) to interact with immune cells including Kupffer cells and neutrophils, and facilitate the immune cell adhesion via elevating the adhesion molecule expression (Sele and Selp) during sepsis (Fig. 5D), which can provide precise drug targets for early treatment of sepsis.

ATF4 inhibition alleviated CLP-induced acute liver dysfunction and prolonged the survival of septic mice

In the present study, the TFs underlying the differential gene expression in endothelial cells, Kupffer cells, neutrophils, and monocytes/monocyte-derived macrophages during the sepsis-induced acute liver dysfunction were identified (Fig. S6A–D), and the representative results were verified via immunofluorescence. As shown in Fig. S5E and F, a rapid increase of ATF4 and

Statistical differences between groups were assessed using one-way ANOVA for (B)–(G). Statistical differences between groups were assessed using a *t* test for (K)–(M). Statistical differences between groups were analysed using the log-rank test for (O). ALT, alanine aminotransferase; AST, aspartate aminotransferase; ATF4, activating transcription factor 4; CLP, caecal ligation and puncture; DBIL, direct bilirubin; GGT, glutamyl-transpeptidase; GLO, globulin; ISR, ISRIB; ISRIB, integrated stress response inhibitor; MDA, malondialdehyde; MLR, monocyte-to-lymphocyte ratio; MPO, myeloperoxidase; NLR, neutrophil-to-lymphocyte ratio; PLT, platelet counts; TBA, total bile acid.

NF-κB1 expression in endothelial cells were observed 6 h after CLP when compared with that of healthy controls. Subsequently, the ATF4 and NF-κB1 expression declined at 24 h after CLP compared with that at 6 h after CLP. In addition, the Fosl1 expression in Kupffer cells increased with pathological progression (Fig. S6G).

Among these TFs, ATF4 was identified as the hallmark TF in endothelial cells, Kupffer cells, neutrophils, and monocytes/monocyte-derived macrophages at 6 h after sepsis (Fig. S6A–D). These results suggested that ATF4 activated the endothelial and immune cells associated with the hyperinflammatory response, which contributed to the pathogenesis of CLP-induced acute liver dysfunction in the early stage of sepsis; thus, we hypothesised that the inhibition of ATF4 would attenuate the CLP-induced acute liver dysfunction.

ISRIB, a specific small-molecule inhibitor, can suppress the upregulation of ATF4 and decrease nuclear translocation of ATF4.[42] As shown in Fig. 6A and B, the plasma ALT and AST activities were used as biomarkers of liver dysfunction. CLP significantly elevated the plasma AST and ALT activities compared with those in the normal controls, which indicated liver dysfunction in the CLP group. ISRIB administration significantly reduced the other liver dysfunction biomarkers, including direct bilirubin, glutamyl-transpeptidase, globulin, total bile acid, and albumin-to-globulin ratio, in plasma compared with those of the CLP group (Fig. 6D–G and Fig. S6H). Notably, ISRIB administration alone showed no significant differences in the liver dysfunction biomarkers compared with those in the normal controls (p >0.05), which suggested that ISRIB exerted no acute toxicity at the administered dosage.

Elevated neutrophil-to-lymphocyte ratio and monocyte-to-lymphocyte ratio have been proposed as indicators of a systemic inflammatory response.[43,44] Recently, research has exhibited that severe cases of COVID-19 tended to have a higher neutrophil-to-lymphocyte ratio.[43,44] Blood analysis demonstrated that CLP significantly reduced the platelet count and increased the neutrophil-to-lymphocyte ratio and monocyte-to-lymphocyte ratio, which were markedly reversed by ISRIB treatment (Fig. 6H–J). These results indicated that ISRIB treatment could decrease the systemic inflammatory response during the early stage of sepsis. In addition, the ISRIB treatment significantly elevated the number of lymphocytes and lymphocyte percentage compared with that of the CLP group (Fig. S6J and I), which suggests that ISRIB treatment promotes immune cell survival and maintains immune function during sepsis.

Hepatic lipid peroxidation and neutrophil infiltration are detected by quantifying the MDA content and MPO activity.[13,45] As shown in Fig. 6K, a significantly higher hepatic MDA content was observed in septic model mice compared with that in the normal control, and the mice administered with ISRIB exhibited a significantly decreased MDA content compared with that in the sepsis model mice. ISRIB administration significantly inhibited neutrophil infiltration in treated mice compared with that in untreated sepsis model mice (Fig. 6L). In comparison with that in the normal control, significantly higher hepatic IL-6 level was indicated in the sepsis model mice treated with vehicle. ISRIB treatment significantly diminished the hepatic IL-6 level compared with that in the sepsis control (Fig. 6M).

As shown in Fig. 6N and Fig. S6K, we observed a marked liver injury including cell swelling, inflammatory cell infiltration, and tissue architecture disruption in sepsis model mice, whereas ISRIB administration exhibited a significant improvement in the pathological injury. To further explore the therapeutic potential of ISRIB, the effect of ISRIB administration on the survival of CLP-induced septic mice was examined. The CLP induced the death in mice, and a single-dose administration of ISRIB significantly improved the survival of CLP mice from 52 to 75% (p <0.05; Fig. 6O).

Discussion

Liver tissue is composed of multiple nonparenchymal cell lineages including neutrophils, endothelial cells, and Kupffer cells, all of which are essential contributors to uncontrolled local inflammation during sepsis exposure, causing intractable liver injury. The characteristics of gene heterogeneity differentiate these major cell types and define multiple subpopulations with distinct functions. Therefore, resolving the dynamic changes of the transcriptome at the single-cell level during disease progression is critical to understanding the intrinsic mechanisms of sepsis-induced acute liver dysfunction, which is of great significance for improving disease diagnosis and intervention. In the current study, scRNA-seq was used to characterise the dynamic cellular and molecular signatures along the disease course, and the key findings were validated in a CLP-induced sepsis model.

This study provides new insights into the role of endothelial cell states in the pathogenesis of sepsis-induced acute liver dysfunction. The number of hepatic endothelial cells was observed to be decreasing in a time-dependent manner during the progression of sepsis, which is consistent with a recent study in an endotoxemia model.[46] We speculated that the weak cellular adhesion and high apoptosis revealed by scRNA-seq might be the main cause of the sharp reduction in the number of endothelial cells, which then triggers interstitial oedema and consecutively worsens liver hypoperfusion, accelerating liver injury.[47] In addition, pieces of clinical evidence also indicate that endothelial damage occurred during the pathogenesis of sepsis and COVID-19 infection,[48,49] leading to an increased circulating endothelial cell, endothelium barrier dysfunction,[50] and subsequently organ injury including acute liver injury.[51] These experimental and clinical results suggest the importance of endothelium protection in early sepsis.

Endothelial cells could undergo dynamic phenotypic switching when exposed to various environments.[51,52] We found that hepatic endothelial cells could be reprogrammed into cells displaying pro-inflammatory phenotype during sepsis, as ECs-3 and ECs-4 were observed to be in different pro-inflammatory states reflected by their inflammation-related gene expression profiles. Particularly, this result indicated that ECs-3 may play an important role in the adaptive immune response to infection as antigen-presenting cells, as reported in other studies as well.[53,54] NOD1 is an important and well-characterised member of the NOD-like receptor family, which was demonstrated to enhance antigen-presenting ability of liver sinusoidal endothelial cells during viral infection.[55] We also found *Nod1* to be highly expressed in ECs-3. Thus, NOD1 is likely to be involved in the antigen-presenting activity of ECs-3 during sepsis. In addition, ECs-4 was elicited to be in an endothelial activation/dysfunction state owing to a high expression of endothelial activation/dysfunction markers, including Vcam-1, Icam-1, Selp, Sele, Serpine1, and Fgf23.[18,21,22,56] The activated ECs-4 could interact with neutrophils and exacerbate sepsis-induced acute liver dysfunction by cell-to-cell communication.[13] We found that the Csf3r/Csf3 axis

was only presented in the interactions between neutrophils and activated ECs-4, suggesting that blocking the Csf3r/Csf3 axis may be a potential therapeutic avenue in treating sepsis-induced acute liver dysfunction, but this still requires further research.

Kupffer cells are a critical component of the mononuclear phagocytic system and are central to both the hepatic and systemic response to pathogens.[57] Previous studies have indicated that the polarisation of Kupffer cells is recognised as a critical mediator of liver injury.[3,58] Traditionally, Kupffer cells are polarised into distinct phenotypes, M1 (inflammatory) and M2 (anti-inflammatory), depending on the local microenvironment.[58] In this study, beyond the quiescent KCs-1, the inflammatory Kupffer cells including KCs-2, KCs-3, and KCs-4 were also identified, and the expression of M1 and M2 Kupffer cell markers were subsequently determined in all five Kupffer cell subpopulations. We found that all the subpopulations showed dual expression of pro-inflammatory genes and anti-inflammatory genes. Specifically, although IL-10, Arg1, and Chil3 were reported as classical M2 Kupffer cell markers,[59] the inflammatory Kupffer cell subpopulations, namely KCs-2, KCs-3, and KCs-4, with their high expression of pro-inflammatory genes, also exhibited a high level of expression of IL-10, Arg1, and Chil3. Similar phenomena have also been reported in other studies.[60,61] Altogether, these results indicated that Kupffer cells are highly plastic and could undergo a broad spectrum of transcriptomic activation states under a complex hepatic microenvironment and cannot be simply classified into either M1 or M2 polarisation. A more comprehensive classification system based on recent research is required to describe the activation states of Kupffer cells *in vivo*.

As the solution injected into the abdominal cavity could flow out though the wound, we do not have the data to evaluate the therapeutic effect if ISRIB was to be given after CLP. Our results indicate that the ATF-4 activation occurs at 6 h after CLP and returns to normal at 24 h after CLP. We speculate that ISRIB exhibits therapeutic effect if ISRIB is given early (<6 h) and that the therapeutic effect might decrease with a prolonged administration time.

In summary, our study presents the dynamic transcriptomic landscape of major nonparenchymal cells at single-cell resolution, in which we identified the significant alterations and heterogeneity of hepatic nonparenchymal cell subsets during sepsis. Importantly, we identified the endothelial cell and neutrophil subsets that are associated with acute liver dysfunction during sepsis progression and explored the therapeutic effect of ATF4 inhibition. Overall, these results uncovered potential mechanisms and promising therapeutic targets for the prevention and treatment of sepsis-induced acute liver dysfunction and other liver-related diseases.

Abbreviations

ALT, alanine aminotransferase; AP-1, activator protein 1; AST, aspartate aminotransferase; ATF4, activating transcription factor 4; CLP, caecal ligation and puncture; CLP-24h, 24 h after CLP; CLP-6h, 6 h after CLP; DBIL, direct bilirubin; ECs-1 to ECs-4, endothelial cell subclusters 1 to 4; GGT, glutamyl-transpeptidase; GLO, globulin; ISRIB, integrated stress response inhibitor; KCs-1 to KCs-4, Kupffer cell subclusters 1 to 4; KEGG, Kyoto Encyclopedia of Genes and Genomes; MDA, malondialdehyde; MPO, myeloperoxidase; Neu-1 to Neu-3, neutrophil subclusters 1 to 3; NOD, nucleotide oligomerisation domain; PLT, platelet counts; SCENIC, single-cell regulatory network inference and clustering; scRNA-seq, single-cell RNA sequencing; T/NK, T/natural killer; TBA, total bile acid; TF, transcription factor; TNF, tumour necrosis factor; tSNE, t-distributed stochastic neighbour embedding; UMAP, uniform manifold approximation and projection.

Financial support

This work was supported by the National Natural Science Foundation of China (82170229) and Foundation Strengthening Program Technology Fund Project (2019-JCJQ-JJ-164).

Conflicts of interest

The authors declare no conflict of interest.

Please refer to the accompanying ICMJE disclosure forms for further details.

Authors' contributions

Designed the study: CG, YY, ZH. Performed the experiments and collected the data: CG, RC, XY. Analysed the data: CG, XY, RC, WY, YR, WQ, YG, LM, ZX, YS, ZJ. Prepared the manuscript: LM, CG, RC, XY.

Data availability statement

The data that support the findings of this study are available from the corresponding authors upon reasonable request.

Acknowledgements

The authors thank Xiaohua Yao in OE Biotech Co., Ltd. (Shanghai, China), for her assistance with the data analysis.

Supplementary data

Supplementary data to this article can be found online at https://doi.org/10.1016/j.jhepr.2023.100718.

References

Author names in bold designate shared co-first authorship.

[1] Li H, Liu L, Zhang D, Xu J, Dai H, Tang N, et al. SARS-CoV-2 and viral sepsis: observations and hypotheses. Lancet 2020;395:1517–1520.
[2] Wang D, Yin Y, Yao Y. Advances in sepsis-associated liver dysfunction. Burns Trauma 2014;2:97–105.
[3] Sun J, Zhang J, Wang X, Ji F, Ronco C, Tian J, et al. Gut–liver crosstalk in sepsis-induced liver injury. Crit Care 2020;24:614.
[4] **Choi H**, **Kim Y**, Mirzaaghasi A, Heo J, Kim YN, Shin JH, et al. Exosome-based delivery of super-repressor IκBα relieves sepsis-associated organ damage and mortality. Sci Adv 2020;6:eaaz6980.
[5] Dar WA, Sullivan E, Bynon JS, Eltzschig H, Ju C. Ischaemia reperfusion injury in liver transplantation: cellular and molecular mechanisms. Liver Int 2019;39:788–801.
[6] Ramachandran P, Dobie R, Wilson-Kanamori JR, Dora EF, Henderson BEP, Luu NT, et al. Resolving the fibrotic niche of human liver cirrhosis at single-cell level. Nature 2019;575:512–518.
[7] **Yao R-Q**, **Li Z-X**, Wang L-X, Li Y-X, Zheng L-Y, Dong N, et al. Single-cell transcriptome profiling of the immune space-time landscape reveals dendritic cell regulatory program in polymicrobial sepsis. Theranostics 2022;12:4606–4628.
[8] Terkelsen MK, Bendixen SM, Hansen D, Scott EAH, Moeller AF, Nielsen R, et al. Transcriptional dynamics of hepatic sinusoid-associated cells after liver injury. Hepatology 2020;72:2119–2133.
[9] Zheng C, Zheng L, Yoo J-K, Guo H, Zhang Y, Guo X, et al. Landscape of infiltrating T cells in liver cancer revealed by single-cell sequencing. Cell 2017;169:1342–1356.e1316.
[10] Xiong X, Kuang H, Liu T, Lin JD. A single-cell perspective of the mammalian liver in health and disease. Hepatology 2020;71:1467–1473.
[11] Li J-Y, Ren C, Wang L-X, Yao R-Q, Dong N, Wu Y, et al. Sestrin2 protects dendrite cells against ferroptosis induced by sepsis. Cell Death Dis 2021;12:834.
[12] **Song X**, **Shang P**, **Sun Z**, Lu M, You G, Yan S, et al. Therapeutic effect of yttrium oxide nanoparticles for the treatment of fulminant hepatic failure. Nanomedicine 2019;14:2519–2533.

[13] **Xiao Y**, **Ren C**, **Chen G**, Shang P, Song X, You G, et al. Neutrophil membrane-mimicking nanodecoys with intrinsic anti-inflammatory properties alleviate sepsis-induced acute liver injury and lethality in a mouse endotoxemia model. Mater Today Bio 2022;14:100244.
[14] **Xie X**, **Shi Q**, Wu P, Zhang X, Kambara H, Su J, et al. Single-cell transcriptome profiling reveals neutrophil heterogeneity in homeostasis and infection. Nat Immunol 2020;21:1119–1133.
[15] Ye D, Yang K, Zang S, Lin Z, Chau H-T, Wang Y, et al. Lipocalin-2 mediates non-alcoholic steatohepatitis by promoting neutrophil-macrophage crosstalk via the induction of CXCR2. J Hepatol 2016;65:988–997.
[16] Khare R, May SM, Vetrini F, Weaver EA, Palmer D, Rosewell A, et al. Generation of a Kupffer cell-evading adenovirus for systemic and liver-directed gene transfer. Mol Ther 2011;19:1254–1262.
[17] Crouch EE, Doetsch F. FACS isolation of endothelial cells and pericytes from mouse brain microregions. Nat Protoc 2018;13:738–751.
[18] Skibsted S, Jones AE, Puskarich MA, Arnold R, Sherwin R, Trzeciak S, et al. Biomarkers of endothelial cell activation in early sepsis. Shock 2013;39:427–432.
[19] Kuriakose T, Man SM, Malireddi RKS, Karki R, Kesavardhana S, Place DE, et al. ZBP1/DAI is an innate sensor of influenza virus triggering the NLRP3 inflammasome and programmed cell death pathways. Sci Immunol 2016;1:aag2045.
[20] Rebsamen M, Heinz LX, Meylan E, Michallet M-C, Schroder K, Hofmann K, et al. DAI/ZBP1 recruits RIP1 and RIP3 through RIP homotypic interaction motifs to activate NF-κB. EMBO Rep 2009;10:916–922.
[21] Silswal N, Touchberry CD, Daniel DR, McCarthy DL, Zhang S, Andresen J, et al. FGF23 directly impairs endothelium-dependent vasorelaxation by increasing superoxide levels and reducing nitric oxide bioavailability. Am J Physiol Endocrinol Metab 2014;307:E426–E436.
[22] Teijeira Á, Palazón A, Garasa S, Marré D, Aubá C, Rogel A, et al. CD137 on inflamed lymphatic endothelial cells enhances CCL21-guided migration of dendritic cells. FASEB J 2012;26:3380–3392.
[23] Liu S, Zheng F, Cai Y, Zhang W, Dun Y. Effect of long-term exercise training on lncRNAs expression in the vascular injury of insulin resistance. J Cardiovasc Transl Res 2018;11:459–469.
[24] **Deng C**, **Zhao L**, **Yang Z**, Shang J-J, Wang C-Y, Shen M-Z, et al. Targeting HMGB1 for the treatment of sepsis and sepsis-induced organ injury. Acta Pharmacol Sin 2022;43:520–528.
[25] **Bao G-Q**, **He L**, Lee D, D'Angelo J, Wang H-C. An ongoing search for potential targets and therapies for lethal sepsis. Mil Med Res 2015;2:20.
[26] Dey S, Baird TD, Zhou D, Palam LR, Spandau DF, Wek RC. Both transcriptional regulation and translational control of ATF4 are central to the integrated stress response. J Biol Chem 2010;285:33165–33174.
[27] Li C. The role of endoplasmic reticulum stress in the development of fibrosis in Crohn's disease. ERHM 2018;3:33–41.
[28] Pandey VK, Mathur A, Khan MF, Kakkar P. Activation of PERK-eIF2α-ATF4 pathway contributes to diabetic hepatotoxicity: attenuation of ER stress by Morin. Cell. Signal 2019;59:41–52.
[29] Zhang T, Wu Y, Hu Z, Xing W, Kun LV, Wang D, et al. Small-molecule integrated stress response inhibitor reduces susceptibility to postinfarct atrial fibrillation in rats via the inhibition of integrated stress responses. J Pharmacol Exp Ther 2021;378:197–206.
[30] Krukowski K, Nolan A, Frias ES, Boone M, Ureta G, Grue K, et al. Small molecule cognitive enhancer reverses age-related memory decline in mice. eLife 2020;9:e62048.
[31] Akhtar S, Hartmann P, Karshovska E, Rinderknecht F-A, Subramanian P, Gremse F, et al. Endothelial hypoxia-inducible factor-1α promotes atherosclerosis and monocyte recruitment by upregulating microRNA-19a. Hypertension 2015;66:1220–1226.
[32] Ma K, Xu H, Zhang J, Zhao F, Liang H, Sun H, et al. Insulin-like growth factor-1 enhances neuroprotective effects of neural stem cell exosomes after spinal cord injury via an miR-219a-2-3p/YY1 mechanism. Aging 2019;11:12278–12294.
[33] **Lin J**, **He Y**, Wang B, Xun Z, Chen S, Zeng Z, et al. Blocking of YY1 reduce neutrophil infiltration by inhibiting IL-8 production via the PI3K–Akt–mTOR signaling pathway in rheumatoid arthritis. Clin Exp Immunol 2019;195:226–236.
[34] **Rehnström M**, **Frederiksen SD**, Ansar S, Edvinsson L. Transcriptome profiling revealed early vascular smooth muscle cell gene activation following focal ischemic stroke in female rats – comparisons with males. BMC Genomics 2020;21:883.
[35] Zhang CY, Dong X, Gao J, Lin W, Liu Z, Wang Z. Nanoparticle-induced neutrophil apoptosis increases survival in sepsis and alleviates neurological damage in stroke. Sci Adv 2019;5:eaax7964.
[36] Liu K, Wang F-S, Xu R. Neutrophils in liver diseases: pathogenesis and therapeutic targets. Cell Mol Immunol 2021;18:38–44.
[37] Blériot C, Ginhoux F. Understanding the heterogeneity of resident liver macrophages. Front Immunol 2019;10:2694.
[38] Wu Y, Hao C, Liu X, Han G, Yin J, Zou Z, et al. MitoQ protects against liver injury induced by severe burn plus delayed resuscitation by suppressing the mtDNA-NLRP3 axis. Int Immunopharmacol 2020;80:106189.
[39] **Engblom C**, **Pfirschke C**, Pittet MJ. The role of myeloid cells in cancer therapies. Nat Rev Cancer 2016;16:447–462.
[40] Saha B, Bala S, Hosseini N, Kodys K, Szabo G. Krüppel-like factor 4 is a transcriptional regulator of M1/M2 macrophage polarization in alcoholic liver disease. J Leukoc Biol 2015;97:963–973.
[41] Remmerie A, Martens L, Thoné T, Castoldi A, Seurinck R, Pavie B, et al. Osteopontin expression identifies a subset of recruited macrophages distinct from Kupffer cells in the fatty liver. Immunity 2020;53:641–657.e614.
[42] Chang L, Liu X, Chen J, Liu H, Wang G, Wang G, et al. Attenuation of activated eIF2α signaling by ISRIB treatment after spinal cord injury improves locomotor function. J Mol Neurosci 2022;72:585–597.
[43] Liu Y, Du X, Chen J, Jin Y, Peng L, Wang HHX, et al. Neutrophil-to-lymphocyte ratio as an independent risk factor for mortality in hospitalized patients with COVID-19. J Infect 2020;81:e6–e12.
[44] Mazza MG, Lucchi S, Rossetti A, Clerici M. Neutrophil-lymphocyte ratio, monocyte-lymphocyte ratio and platelet-lymphocyte ratio in non-affective psychosis: a meta-analysis and systematic review. World J Biol Psychiatry 2020;21:326–338.
[45] Chen G, Song X, Wang B, You G, Zhao J, Xia S, et al. Carboxyfullerene nanoparticles alleviate acute hepatic injury in severe hemorrhagic shock. Biomaterials 2017;112:72–81.
[46] Sun X, Wu J, Liu L, Chen Y, Tang Y, Liu S, et al. Transcriptional switch of hepatocytes initiates macrophage recruitment and T cell suppression in endotoxemia. J Hepatol 2022;77:436–452.
[47] Aird WC. Endothelial cell heterogeneity. Crit Care Med 2003;31:S221–S230.
[48] Tapia P, Gatica S, Cortés-Rivera C, Otero C, Becerra A, Riedel CA, et al. Circulating endothelial cells from septic shock patients convert to fibroblasts are associated with the resuscitation fluid dose and are biomarkers for survival prediction. Crit Care Med 2019;47:942–950.
[49] Xu S-W, Ilyas I, Weng J-P. Endothelial dysfunction in COVID-19: an overview of evidence, biomarkers, mechanisms and potential therapies. Acta Pharmacol Sin 2022:1–15.
[50] Opal SM, van der Poll T. Endothelial barrier dysfunction in septic shock. J Intern Med 2015;277:277–293.
[51] **Poisson J**, **Lemoinne S**, Boulanger C, Durand F, Moreau R, Valla D, et al. Liver sinusoidal endothelial cells: physiology and role in liver diseases. J Hepatol 2017;66:212–227.
[52] Joffre J, Hellman J, Ince C, Ait-Oufella H. Endothelial responses in sepsis. Am J Respir Crit Care Med 2020;202:361–370.
[53] Jin Y, Ji W, Yang H, Chen S, Zhang W, Duan G. Endothelial activation and dysfunction in COVID-19: from basic mechanisms to potential therapeutic approaches. Signal Transduct Target Ther 2020;5:293.
[54] **Paik DT**, **Tian L**, **Williams IM**, Rhee S, Zhang H, Liu C, et al. Single-cell RNA sequencing unveils unique transcriptomic signatures of organ-specific endothelial cells. Circulation 2020;142:1848–1862.
[55] Huang S, Zou S, Chen M, Gao X, Chen L, Yang X, et al. Local stimulation of liver sinusoidal endothelial cells with a NOD1 agonist activates T cells and suppresses hepatitis B virus replication in mice. J Immunol 2018;200:3170–3179.
[56] **Chen G**, **Zhao J**, Yin Y, Wang B, Liu Q, Li P, et al. C-type natriuretic peptide attenuates LPS-induced endothelial activation: involvement of p38, Akt, and NF-κB pathways. Amino Acids 2014;46:2653–2663.
[57] Dixon LJ, Barnes M, Tang H, Pritchard MT, Nagy LE. Kupffer cells in the liver. Compr Physiol 2013;3:785–797.
[58] Abdullah Z, Knolle PA. Liver macrophages in healthy and diseased liver. Pflügers Arch 2017;469:553–560.
[59] Sica A, Mantovani A. Macrophage plasticity and polarization: in vivo veritas. J Clin Invest 2012;122:787–795.
[60] Zhao G, Lu H, Chang Z, Zhao Y, Zhu T, Chang L, et al. Single-cell RNA sequencing reveals the cellular heterogeneity of aneurysmal infrarenal abdominal aorta. Cardiovasc Res 2020;117:1402–1416.
[61] Tacke F. Targeting hepatic macrophages to treat liver diseases. J Hepatol 2017;66:1300–1312.

Journal of Hepatology, Volume 5

Supplemental information

Time-resolved single-cell transcriptomics reveals the landscape and dynamics of hepatic cells in sepsis-induced acute liver dysfunction

Gan Chen, Chao Ren, Yao Xiao, Yujing Wang, Renqi Yao, Quan Wang, Guoxing You, Mingzi Lu, Shaoduo Yan, Xiaoyong Zhang, Jun Zhang, Yongming Yao, and Hong Zhou

Time-resolved single-cell transcriptomics reveals the landscape and dynamics of hepatic cells in sepsis-induced acute liver dysfunction

Gan Chen, Chao Ren, Yao Xiao, Yujing Wang, Renqi Yao, Quan Wang, Guoxing You, Mingzi Lu, Shaoduo Yan, Xiaoyong Zhang, Jun Zhang, Yongming Yao, Hong Zhou

Table of contents:

Fig. S1

Fig. S2

Fig. S3

Fig. S4

Fig. S5

Fig. S6

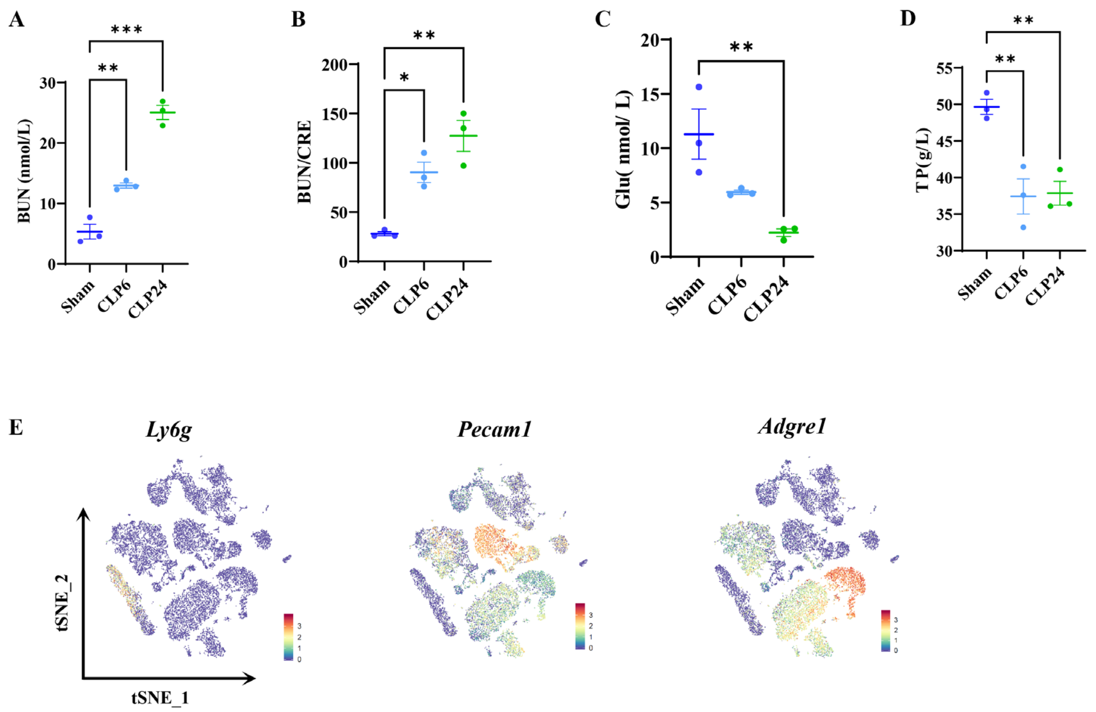
A
B
C
D
BUN (nmol/L)
BUN/CRE
Glu(nmol/ L)
TP(g/L)
Sham
CLP6
CLP24
E
Ly6g
Pecam1
Adgre1
tSNE_2
tSNE_1

F

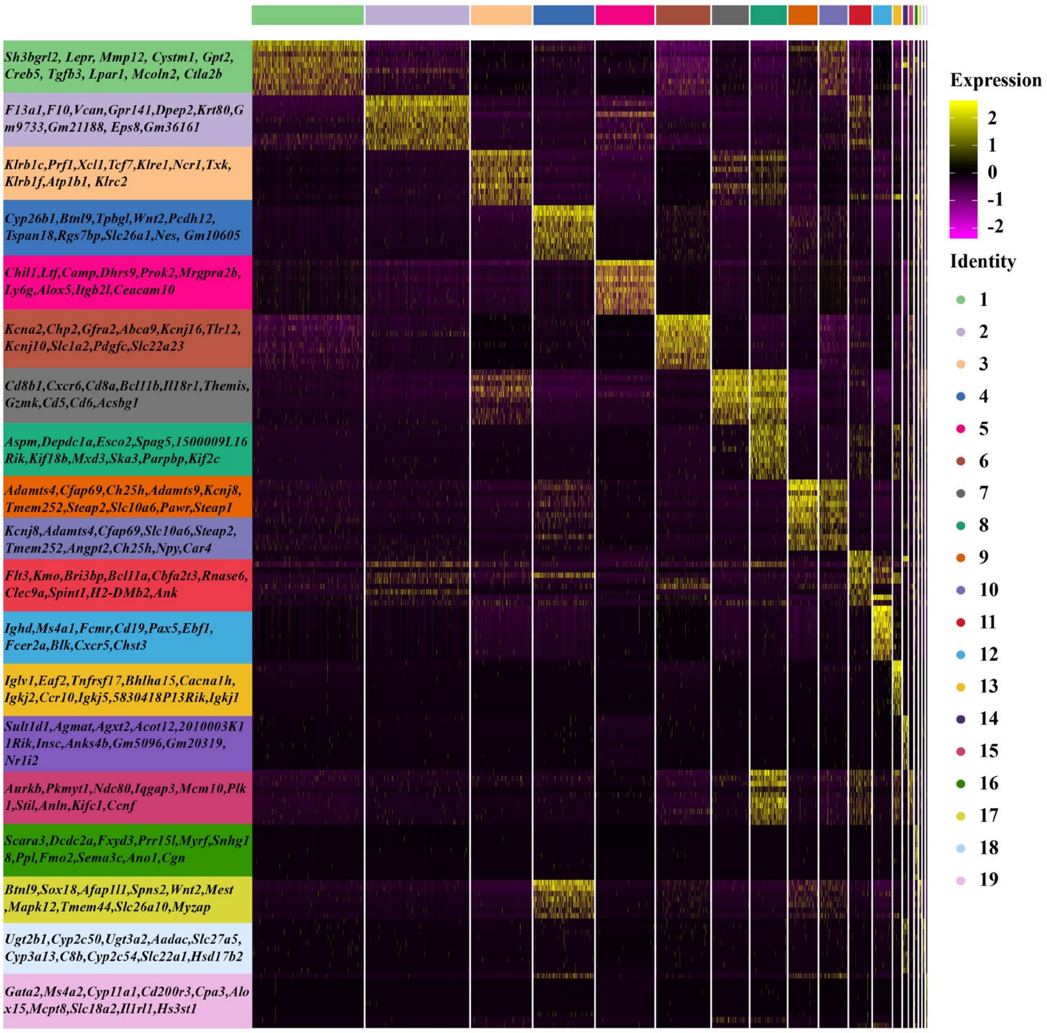

Fig. S1. Biochemical indices in plasma. The levels of blood urea nitrogen (BUN) (A), blood urea nitrogen/creatinine (BUN/CRE) (B), glucose (Glu) (C), total protein (TP) (D) in plasma. (E) The cell markers indicating group identities. (F) Heatmap plots demonstrating expression of specified genes of 19 cell clusters. *p < 0.05; **p < 0.01; ***p < 0.001. Statistical differences between groups were performed by a one-way ANOVA for Fig. S1 A, B, C, and D.

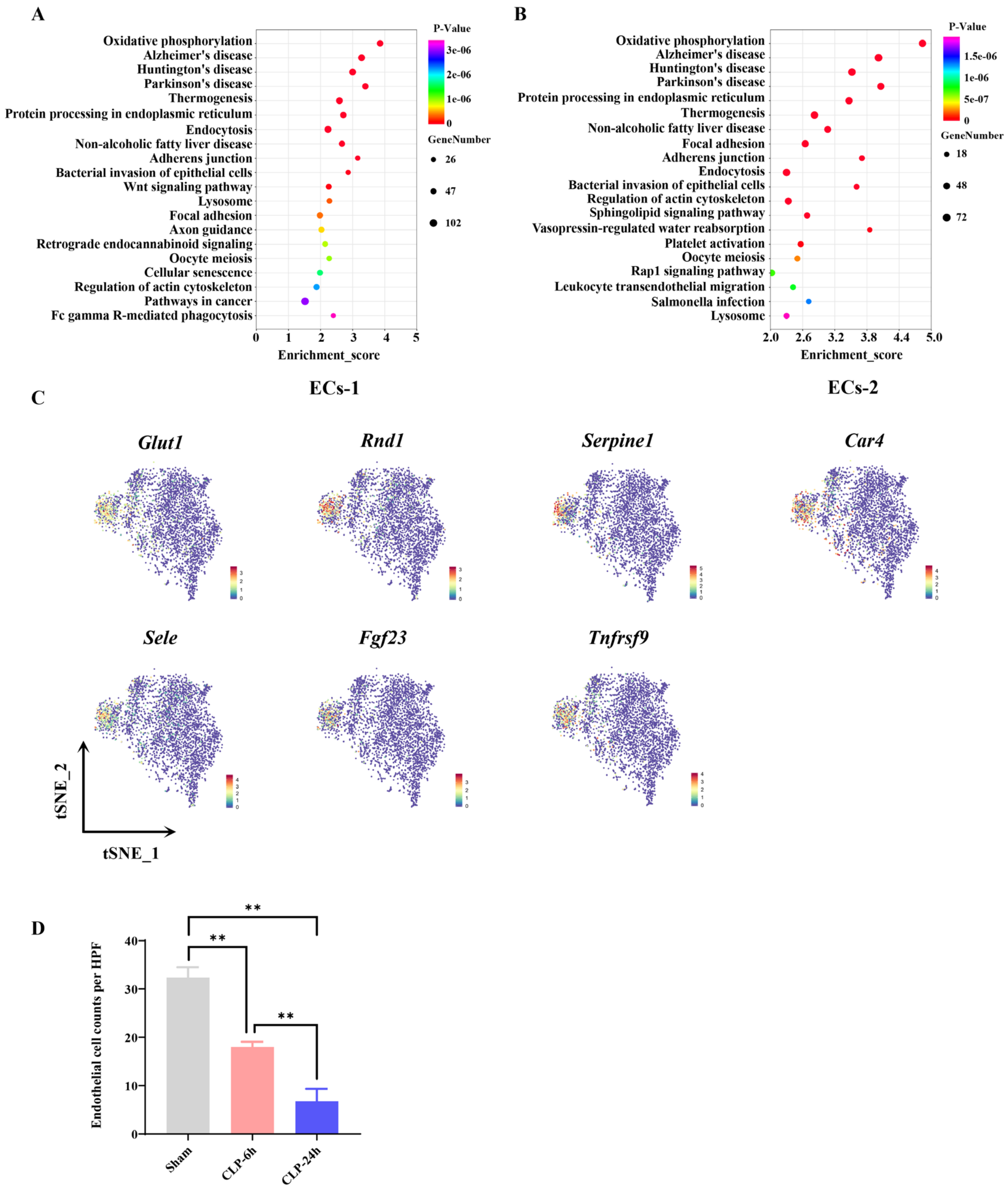

Fig. S2. The enriched pathway of associated with endothelial cell subclusters 1 (A) and 2 (B). Colors indicate significance of enrichment and circle sizes indicate number of genes falling into respective categories. (C) The markers indicating group identities. (D) The average number of neutrophils per high-power field (HPF). $^{**}p < 0.01$. Statistical differences between groups were performed by a one-way ANOVA.

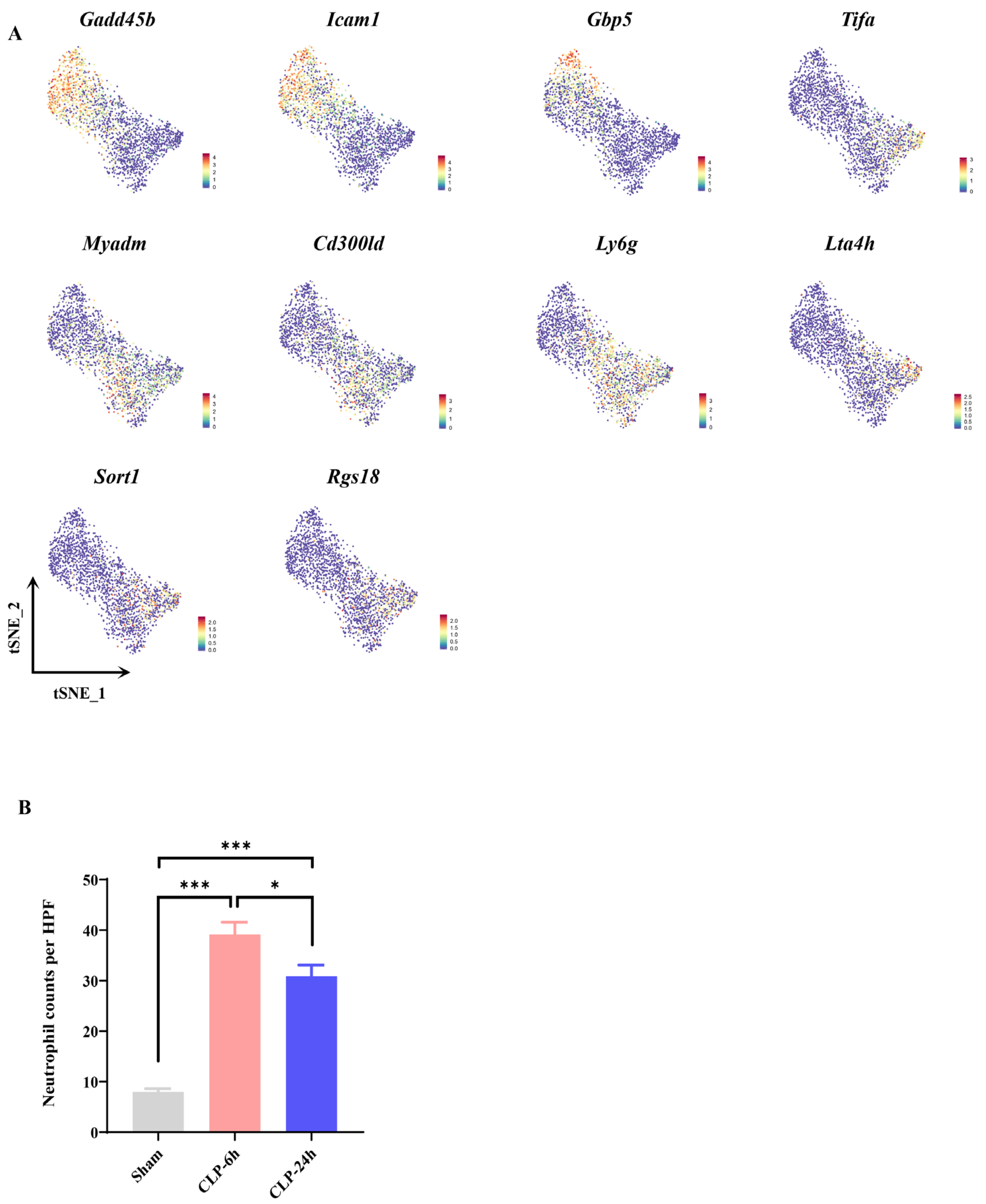

Fig. S3. (A) The markers indicating group identities. (B) The average number of neutrophils (Ly6G positive cells) per high-power field (HPF). *p < 0.05; ***p < 0.001. Statistical differences between groups were performed by a one-way ANOVA.

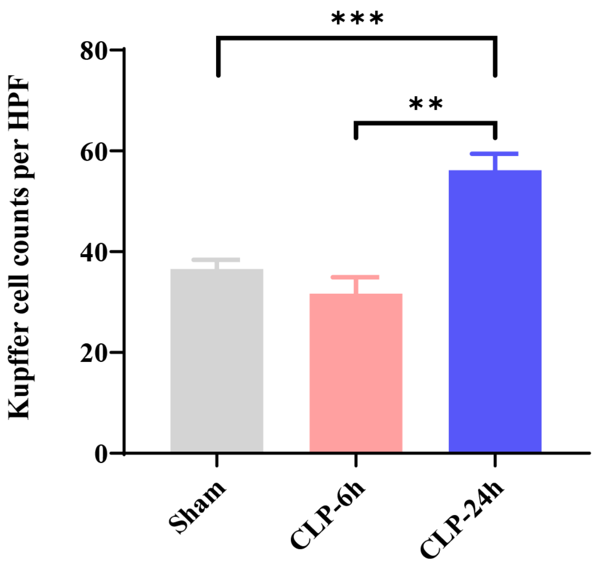

Fig. S4. The average number of Kupffer cells per high-power field (HPF). ***p* < 0.01; ****p* < 0.001. Statistical differences between groups were performed by a one-way ANOVA.

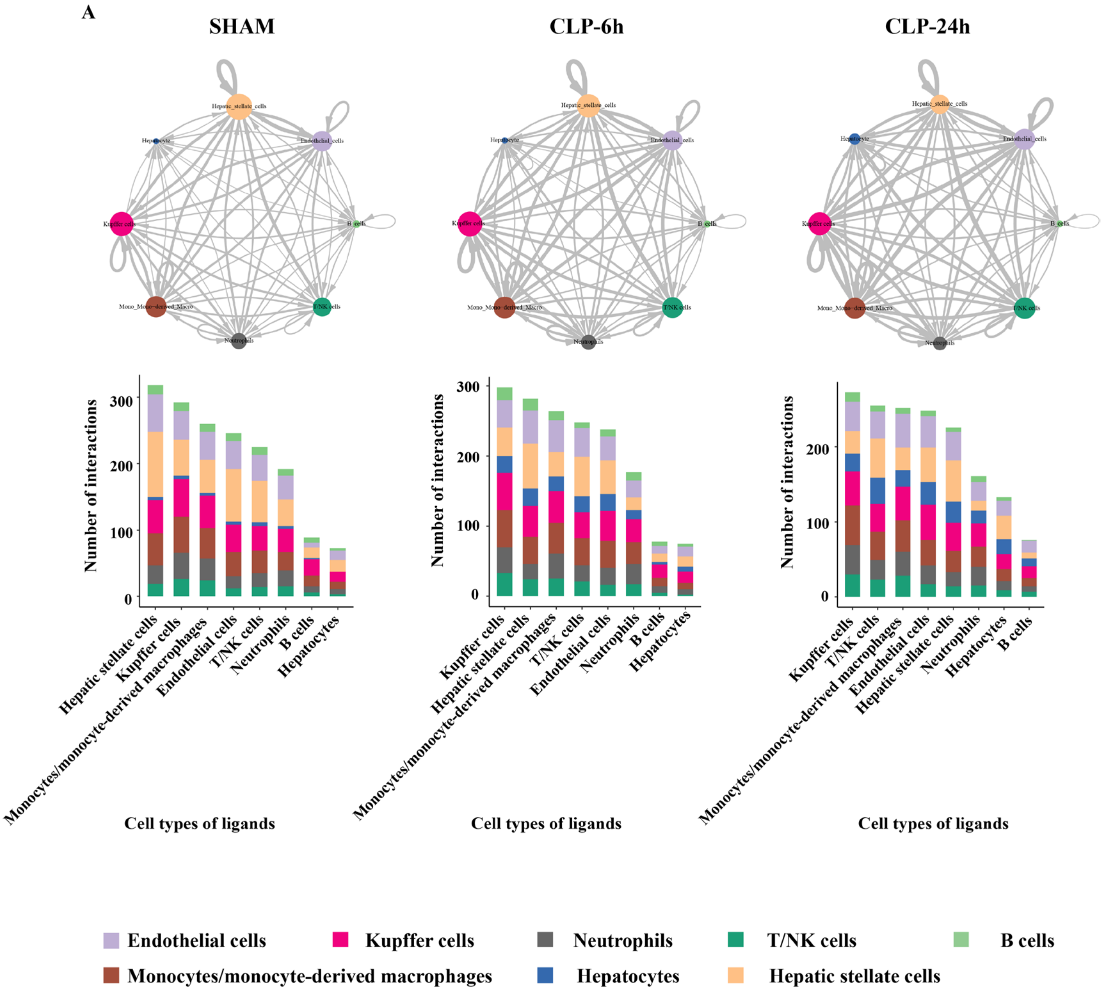

Fig. S5. The cell-cell interaction network and histogram plot shows interaction frequencies between different cell types across the indicated conditions. Colorful nodes represent cell types, and their sizes indicate interaction numbers.

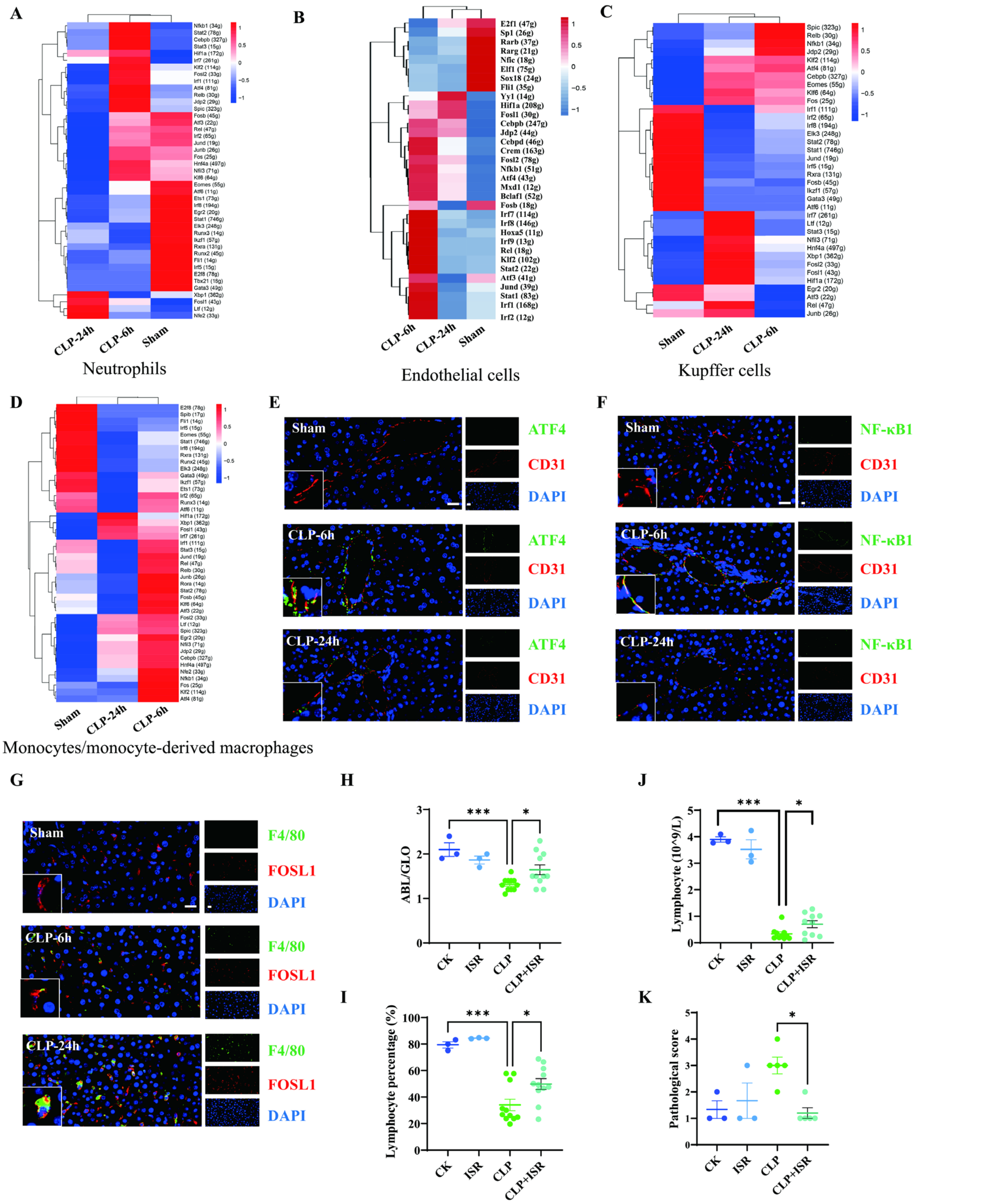

Fig. S6. (A) The specific regulons of each condition for neutrophils revealed by SCENIC analysis. (B) The specific regulons of each condition for endothelial cells revealed by SCENIC analysis. (C) The specific regulons of each condition for Kupffer cells revealed by SCENIC analysis. (D) The specific regulons of each condition for monocytes/monocyte-derived macrophages revealed by SCENIC analysis. Immunofluorescence staining results showing the protein expression of ATF4 (E) and

NF-κB1 (F) in endothelial cells of healthy and septic mouse livers. Scale bars, 20 μm. (G) Immunofluorescence staining results showing the protein expression of FOSL1 in Kuppfer cells of healthy and septic mouse livers. Scale bars, 20 μm. (H) The levels of albumin (ALB)/globulin (GLB) ratio in plasma 24 h after CLP. The levels of lymphocyte percentage (I) and count (J) in blood 24 h after CLP. K. Histologic liver injury scores. ISR: ISRIB, *p < 0.05; * * *p < 0.001. Statistical differences between groups were performed by a one-way ANOVA for Fig. S6 H, J, and I. Statistical differences between groups were performed by t-test for Fig. 6K.
